# Dendritic cell subsets and implications for cancer immunotherapy

**DOI:** 10.3389/fimmu.2024.1393451

**Published:** 2024-06-05

**Authors:** Michael Y. Chen, Felicia Zhang, Simon Peter Goedegebuure, William E. Gillanders

**Affiliations:** ^1^ Department of Surgery, Washington University School of Medicine, St. Louis, MO, United States; ^2^ Alvin J. Siteman Cancer Center at Barnes-Jewish Hospital, Washington University School of Medicine, St. Louis, MO, United States

**Keywords:** dendritic cell (DC), dendritic cell vaccine, dendritic cell subtypes, cancer immune therapy, cancer vaccination, tumor neoantigen, dendritic cell targeting, antigen presentation

## Abstract

Dendritic cells (DCs) play a central role in the orchestration of effective T cell responses against tumors. However, their functional behavior is context-dependent. DC type, transcriptional program, location, intratumoral factors, and inflammatory milieu all impact DCs with regard to promoting or inhibiting tumor immunity. The following review introduces important facets of DC function, and how subset and phenotype can affect the interplay of DCs with other factors in the tumor microenvironment. It will also discuss how current cancer treatment relies on DC function, and survey the myriad ways with which immune therapy can more directly harness DCs to enact antitumor cytotoxicity.

## Introduction

Harnessing the immune system to treat malignancies has become a powerful tool in cancer therapy, with an explosion of FDA-approved immunotherapies in recent years. As primary mediators of cytotoxic activity against tumors, CD8 T cells are the focus of current treatments such as immune checkpoint inhibition (1), CAR-T cell therapies (2), and cancer vaccines (3). The generation of effective CD8 T cell responses, however, is a complex process involving multiple components of the immune system.

Dendritic cells (DCs) play a central role in the orchestration of effective CD8 T cell responses against tumors (4, 5). At the most fundamental level, T cell-mediated anticancer immune responses center around antigen presentation by DCs. This process starts with DC capture of tumor-derived antigens, which are intracellularly loaded onto MHC molecules. These peptide MHC complexes (pMHC) are then transported to the cell surface to prime and activate effector T cells within the tumor-draining lymph node. Whereas antigen loading onto MHC class I molecules on DCs primes CD8 T cells, presentation of antigens by MHC class II molecules can prime CD4 T helper (Th) cells. “CD4 help,” particularly by activated effector memory Th1 cells, enhances priming of CD8 T cells through CD40 signaling on DCs (6, 7). This interaction, in turn, promotes cross-presentation (8), trafficking of T cells to the tumor, and induction of effector function and memory formation (9). Within the tumor microenvironment (TME), cytotoxic T cells recognize their cognate antigen on tumor cells, which ultimately leads to cancer cell death. The subsequent release of tumor antigens and uptake by antigen-presenting cells restarts what is referred to as the “cancer immunity cycle.”

The centrality of DCs in tumor immunity is demonstrated in pre-clinical models where DCs are found to be critical to T cell based immunotherapies (10, 11). In humans, intratumoral dendritic cells are associated with favorable clinical benefit, whereas DC dysfunction is associated with poor survival (12–14). Yet, whereas the importance of DCs in tumor immunity is increasingly evident, the biology of DCs is still not completely understood. DC functional behavior is context-dependent, as DC type, transcriptional program, location, intratumoral factors, and inflammatory milieu all impact whether DCs promote or inhibit an effective T cell response. The following review introduces key facets of DC function, and how subset and phenotype can affect the interplay of DCs with other cells in the tumor microenvironment. Subsequent discussion will consider how current cancer treatment relies on DC function, and survey the myriad ways with which immune therapy can more directly harness DCs to enact antitumor cytotoxicity. Of note, while there is increasing evidence that DCs can be used to target humoral responses (15), B cell or antibody-based therapies are beyond the scope of this review.

## DC antigen presentation and T-cell priming

Given the integral role of dendritic cells in inducing tumor T cell immunity, the method in which DCs acquire and present tumor antigens to T cells is of special interest. Classically, exogenous antigens are taken up by DCs and processed for MHC class II presentation. However, some exogenous antigens are shuttled into the MHC class I presentation pathway in a specialized process known as cross-presentation. Highlighting the importance of this pathway in tumor immunity is the loss of WDFY4, a BEACH-domain containing protein essential for cross-presentation in conventional DCs, which results in failure to both prime CD8 T cells and reject tumor in preclinical models (16).

Another method by which DCs can acquire and present tumor-derived antigens by MHC-I is MHC-dressing (formerly known as “cross-dressing”). MHC-dressing is the process by which a dendritic cell acquires an intact peptide MHC complex (pMHC) from a neighboring cell. pMHC-I transfer can occur both between dendritic cells (17) and, in cancer, between tumor cells and dendritic cells (18, 19). MHC-dressing in preclinical models has been found to be an effective way to induce tumor immunity independently of cross-presentation (18–21). In fact, in the setting of low levels of antigen, MHC-dressing may even be more efficient than cross-priming (20).

Of note, the DC that acquires and processes a tumor antigen may not be the same DC that eventually primes the tumor-specific T cell in the lymph node. Several mechanisms of antigen transfer between different types of DCs have been proposed, including cross-presentation of phagocytosed donor DC fragments, MHC-dressing, and synaptic transfer of antigen-laden vesicles (22). In cases in which direct DC-T cell interaction is either limited due to low cell density or ineffective due to downregulated antigen presentation, antigen transfer between migratory DCs and lymph node resident DCs has been shown to enhance, amplify, and even salvage CD8 T cell activation (23, 24). Moreover, successful T cell activation by traditional monocyte-derived DC vaccines relies heavily on antigen transfer to endogenous DCs (25, 26), although recent preclinical evidence suggests that a DC vaccine consisting of conventional type I dendritic cells (cDC1), may theoretically overcome this requirement by directly engaging host T cells (10).

Boosting tumor cytotoxic T lymphocyte (CTL) activity is key in tumor immune therapy, and therefore MHC-I-mediated antigen presentation is the primary focus. Yet, CD4 T cell help initiated by pMHC-II complexes is critical in promoting effector and memory CD8 T cell responses (7, 17, 27). Conversely, CD8 T cells primed in the absence of CD4 help are functionally impaired, abrogating tumor control (28). While direct CD4 T cell activation through pMHC-II on tumor cells can be effective in select settings (29, 30), DCs are central to mediating CD4 help. Moreover, cell surface MHC-II expression by tumor cells is often downregulated. DCs, after MHC-II engagement with CD4 T cells, are licensed to enable CD8 effector programs via costimulatory and cytokine signals (7, 31). The utility of directly leveraging DC-mediated CD4 help is evidenced to some degree in stage 3 melanoma patients, a small fraction of whom were found to have detectable circulating tumor antigen-specific CD8 T cells after DC vaccination with both MHC-I- and II-restricted epitopes but not with MHC-I-restricted epitopes alone (32).

## Dendritic cell subtypes

Dendritic cells can be divided into subtypes based on function and phenotypic markers. Initially, conventional DCs (cDCs) were distinguished from plasmacytoid DCs (pDCs) based on their ability to directly present antigens to T cells (33). More recently, understanding of transcription factors driving DC differentiation in mice has further reinforced the divisions of DCs and is continually being refined with the advent of new technologies such as single-cell RNA sequencing (34, 35). Broadly speaking, DCs are divided into three main subsets: Type I and II conventional DCs (cDCs) and plasmacytoid DCs (pDCs). These three subsets function collectively to drive adaptive immune responses.

In humans, DC development begins in the bone marrow from hematopoietic stem cells (HSCs) (36). Granulocyte, monocyte, and DC progenitor (GMDPs) gives rise to monocyte and DC progenitors (MDPs) which can give rise to all DC subsets. MDPs lose the ability to differentiate into monocytes when they become common DC progenitors (CDPs). CDPs can either differentiate into plasmacytoid DCs (pDCs) or a circulating pre-conventional DC (pre-cDC) progenitor capable of becoming either type I or II cDCs.

### Type I conventional dendritic cells

In mice, cDC1s are known as BATF3-dependent DCs due to their reliance on a complexing of transcription factors BATF3 and IRF8 to induce AP1-IRF composite elements (AICE)-dependent auto-activation of IRF8 expression. A high IRF8*/*low IRF4 state then drives gene expression of cDC1-specific genes (37). cDC1s express surface markers XCR1, CLEC9A, CADM1, BTLA, and CD26 across species (38). Most commonly, they are identified by CD8α (lymphoid organ resident) or CD103 (peripheral tissue resident) in mice, and CD141 (BDCA-3) in humans (39, 40). cDC1s are a rare subset of cells in human blood and lymphoid tissues representing <0.01% of CD45 cells, although analysis of deceased transplant donors suggest large variation among individuals (41).

Despite their rarity, cDC1s play an integral role in tumor immunity. BATF3-deficient (*Batf3^-/-^*) mice, the first murine model devoid of cDC1s, are unable to reject even highly immunogenic tumors and do not respond to immune checkpoint blockade or adoptive T cell transfer (42). While monocyte-derived DCs (moDCs) can also perform cross-presentation *in vitro*, indirect evidence suggests that cross-presentation by cDC1s is required for T cell priming against tumors *in vivo*. Cross-presentation by moDCs operate under a distinct BATF3-independent transcriptional program (43). MoDCs also do not require BATF3 for their development. This suggests that moDCs and their cross-presentation are not affected in *Batf3^-/-^* mice and are insufficient for tumor rejection. Later transgenic murine models confirm that *in vivo* tumor antigen cross-presentation is performed by cDC1s. A model lacking MHC-I only on cDC1s, but not other subsets, could not reject tumors (31). Furthermore, deletion of WDFY4, a vesicular trafficking gene required for cross-presentation by cDC1s, but not moDCs, abrogated tumor rejection (16). In a cDC1 deficient mouse model, only vaccination with cDC1 (not other DC subsets) leads to tumor regression (10). Taken together, cross-presentation of tumor antigens by cDC1s is a necessary step for mounting T cell-mediated tumor immunity. Analysis of the Cancer Genome Atlas reveals that cDC1 populations correlate with improved survival in a wide range of malignancies (44).

### Type II conventional dendritic cells

cDC2s are identified by surface markers CD11b, CD1c, and SIRPα (CD172α) in mice and humans, and are driven by low levels of IRF8 and IRF4 transcription factors (38). This low level of IRF8 and IRF4 is sufficient to activate the ETS-IRF composite elements (EICE)-dependent program which leads to expression of common cDC genes, but not the cDC1 specific AICE-dependent program (45–47). Given their robust MHC-II antigen presentation capability, cDC2s were originally believed to be responsible for priming CD4 T cells to help in CD8 T cell activation and priming. Primed CD4 T cells subsequently interact with cDC1s via MHC-II molecules, and, through activation of CD40, enhance priming of CD8 T cells (48, 49).

**Figure 1:**
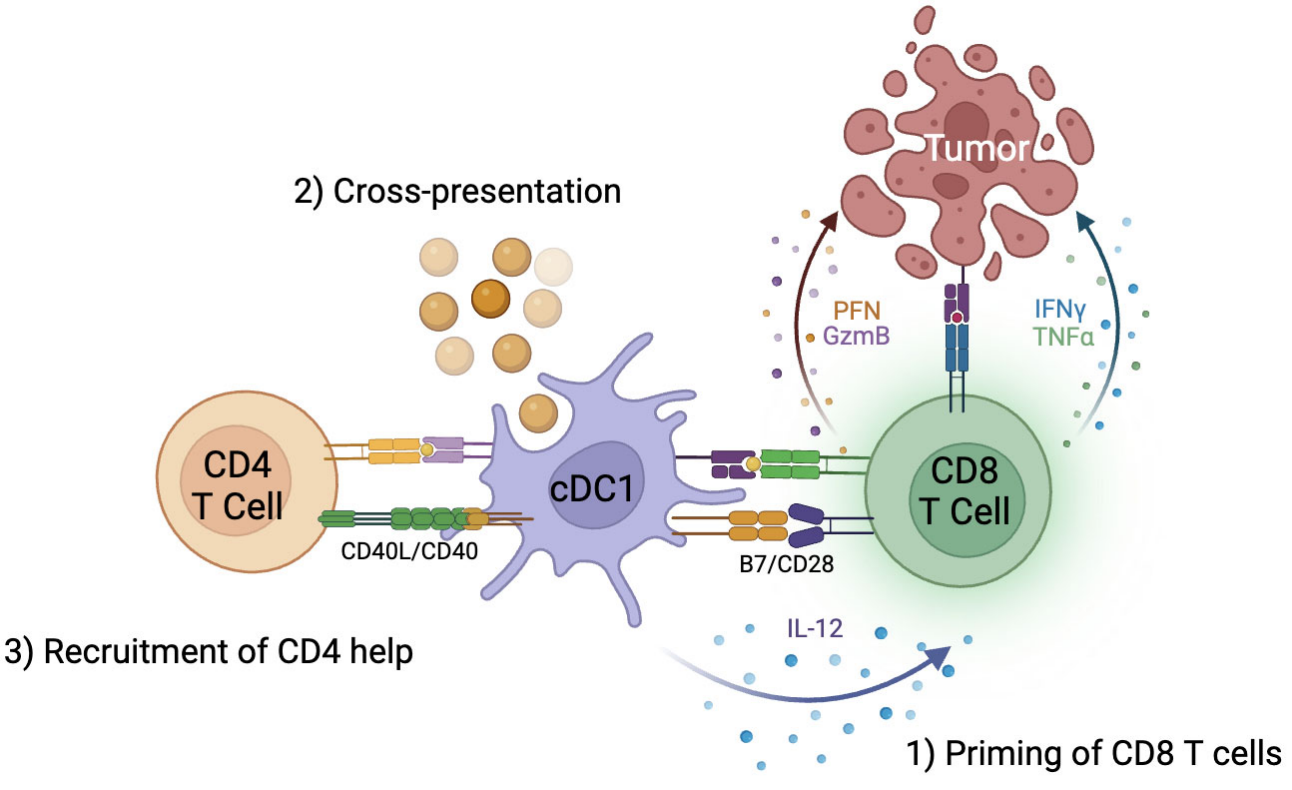
cDC1s are critical to generation of anti-tumor immunity by CD8 T cells. 1) cDC1s are responsible for priming CD8 T cells through B7/CD28 signaling, 2) cross-presentation of exogenous antigens on MHC class I molecules to CD8 T cells, and 3) recruitment of CD4 T cell help and licensing of cDC1 through CD40L/CD40 signaling.

Recent studies in knockout murine models, however, has called this paradigm into question. First, deletion of MHC-II molecules on cDC1s impaired tumor rejection, consistent with the need for interaction of CD4 T cells and cDC1s to effectively prime CD8 T cells. However, lack of MHC-II on cDC1s also reduced CD4 T cell responses. When MHC-II molecules were exclusively expressed on cDC1s, CD4 T cell priming was unaffected (31). Thus, cDC1s are sufficient for priming of CD4 T cells to then license cDC1s to enhance CD8 T cell responses (**Figure 1**).

Heterogeneity of cDC2s is still incompletely understood. Recently, single cell analysis revealed two distinct subpopulations of human cDC2s referred to as DC2 and DC3 (50). Like all conventional DCs, DC3 activate CD8 T cells, but is especially potent in inducing CD8+ CD103+ tissue-resident memory T cells (TRM) and correlated with TRM accumulation in breast cancer (51). These findings suggest the DC3 subset may play an important role in tumor immunity.

### Plasmacytoid DCs

pDCs histologically resemble plasma cells with eccentric nuclei and prominent endoplasmic reticulum. They are known for the ability to secrete inflammatory cytokines such as type I interferon, IL-6, and TNF-α (38). They retain GMDP markers such as CD123 and CD45RA. pDCs are essential mediators of anti-viral immunity, but their role in tumor immunity is less defined (52). Mouse models suggest that tumor associated pDCs may induce tumor immunity when co-administered with TLR7 ligand (53, 54). Clinical data is equivocal in the effect of pDCs on outcomes, although recent high dimensional analysis reveal heterogeneity within what is defined as pDC populations, suggesting variable outcomes could be attributed to different subset populations (55).

### Monocyte-derived DCs

moDCs differentiate from monocytes under inflammatory conditions and represent a heterogeneous group of cells. Since human monocytes express CD11c and MHC-II, it is difficult to distinguish them from true dendritic cells. Dendritic cell surface markers such as CD1c, CD1a, FcεR1, and expression of IRF4 and ZBTB46 help distinguish moDCs from macrophage-like cells (38). Current models suggest moDCs are found primarily at sites of inflammation and have limited migration potential to lymph nodes (56). There was much excitement around applications to immunotherapy given the ability to generate moDCs *in vitro* with GM-CSF and IL-4 culture and maturation through LPS or PGE_2_ stimulation (57), however translation to clinical therapy has had limited success. The intrinsic lack of biological potency compared to the other types of DCs is believed to be the reason for the disappointing performance of moDC-based cancer immunotherapy (58).

## The role of DC in the tumor microenvironment

For DCs to mediate effective tumor immunity, they have to be able to infiltrate and remain functional within the immunosuppressive tumor microenvironment. Within the tumor, NK cells are likely major producers of cDC1 chemoattractants CCL4, CCL5, XCL1, and XCL2 (44, 59, 60) and FLT3L (59). On the contrary, tumor β-catenin signaling disrupts cDC1 recruitment in melanoma (60, 61), and tumor production of factors such as IL-10, IL-6, TGF-β, and PGE_2_ suppresses DC function and maturation (62). When functional, cDC1 produce T cell chemoattractants CXCL9 and CXCL10 (63, 64) as well as IL-12, which drives CD8 T cell effector function (65, 66).

High cDC1 gene signatures, conserved across various solid human tumors (67), is correlated with greater tumor T-cell infiltration (59, 68) and with improved patient response to immune therapy and survival (44, 59, 68). Conversely, in pancreatic ductal adenocarcinoma (PDAC), decreased intratumoral cDC1 numbers are linked to poor immune therapy responsiveness, decreased T-cell priming, and depressed tumor control (69, 70). The few cDC1 that are present in PDAC may, in fact, be tolerogenic. A recent Human Tumor Atlas Network study of 83 PDAC samples across 31 patients found that both cDC1 and cDC2 strongly expressed pro-tumorigenic genes involved in hypoxia and angiogenesis (71). Similarly, dysfunctional cDCs, termed “mregDC,” possess a unique immunoregulatory program upon uptake of tumor antigens and seem to preferentially engage with exhausted, antigen-experienced CD4 T cells (72). mregDC1s in particular are characterized by upregulation of IL4-R expression, that, when targeted with antagonistic antibody, significantly improves IL-12 production and subsequent CTL responses (73).

Given the unique ability of cDC1 to cross-present tumor antigens to CD8 T cells as well as their ability to engage CD4 T cells, they are regarded as the primary DC subset that orchestrates antitumor T cell responses. However, cDC2 have been shown to be a critical driver of tumor immunity in certain contexts. In one preclinical cDC1 diphtheria toxin receptor (DTR) knockout model, intratumoral regulatory T cell (Treg) depletion enhanced cDC2 migration to the tumor-draining lymph node and reversed dysfunction, leading to productive priming and activation of effector CD4 T cells. This step was required for subsequent tumor infiltration and rejection, highlighting that the interaction between Tregs and cDC2s can lead to suppression of tumor immunity (74). Moreover, certain cDC2s, upon acquiring an interferon-stimulating gene (ISG) activation state (ISG+ DCs), are able to MHC-dress with tumor-derived pMHC-1, stimulate CD8 T cells *ex vivo*, and drive tumor control even in the absence of cDC1 (75). Conversely, in the presence of high levels of IL-6 and PGE_2_, intratumoral cDC2s can also be driven toward a pro-tumor phenotype characterized by CD14 expression, upregulation of markers usually associated with tumor-associated macrophages (TAMs), and impaired antigen processing and presentation (76–78).

Similar to that of cDC2s, the role of pDCs in the TME is complex and still poorly understood. Depending on their activation state, they can suppress or promote antitumor immune responses. OX40+ pDC in head and neck squamous cell cancer (HNSCC) and IRF7+ pDC in colon cancer have been associated with increased survival (79, 80), whereas high tumor infiltration of IFN-α-deficient pDC across multiple cancers has been associated with aggressive disease and poor survival (81–83). One proposed mechanism is that tumor-derived TGF-β and other TME suppressive factors suppress IFN-α production by pDC and foster pDC-Treg engagement (83). ICOS-L and IDO expression by pDC then promote Treg proliferation and a subsequently immunosuppressive TME (83–86).

In DCs, metabolism is closely linked to maturation signaling and is therefore a key driver of DC activation or tolerogenicity within the tumor microenvironment. In general, differences in regulation of glycolysis and OXPHOS programs are associated with anti- or pro- inflammatory DC phenotypes. In immature DCs, the AMP-activated kinase (AMPK)/mammalian target of rapamycin (mTOR) axis is suggested to play a crucial role in this balance. AMPK promotes oxidative metabolism and antagonizes mTOR, which upregulates glycolytic pathways after TLR signaling. As AMPK negatively regulates DC activation, its downstream effects include decreased expression of co-stimulatory molecules, reduced CD8 priming capacity, and loss of tumor control (87–89). In moDC, tolerogenic DCs with maturation-resistant phenotypes tend to favor glycolytic pathways whereas CD86+ mature populations tend to favor aerobic OXPHOS, as illustrated by lower p-mTOR:p-AMK ratio (90).

Fatty acid metabolism can also affect DC activation state, in that intracellular lipid accumulation as a response to tumor-derived factors has an inverse effect on DC cross-presentation. One possible mechanism of lipid-dependent DC suppression is that oxidized lipids bind to and inactivate chaperone protein HSP70, leading to accumulation of pMHC in lysosomes instead of on the cell surface (91, 92). Both inhibition of lipid uptake and reduction of fatty acid synthesis in dendritic cells can improve DC (and subsequent T cell) function, even leading to improved vaccine-mediated tumor control in murine tumor models (93). Further intricacies of the relationship between DC metabolism and activation state is expertly discussed elsewhere (94–96).

**Figure 2:**
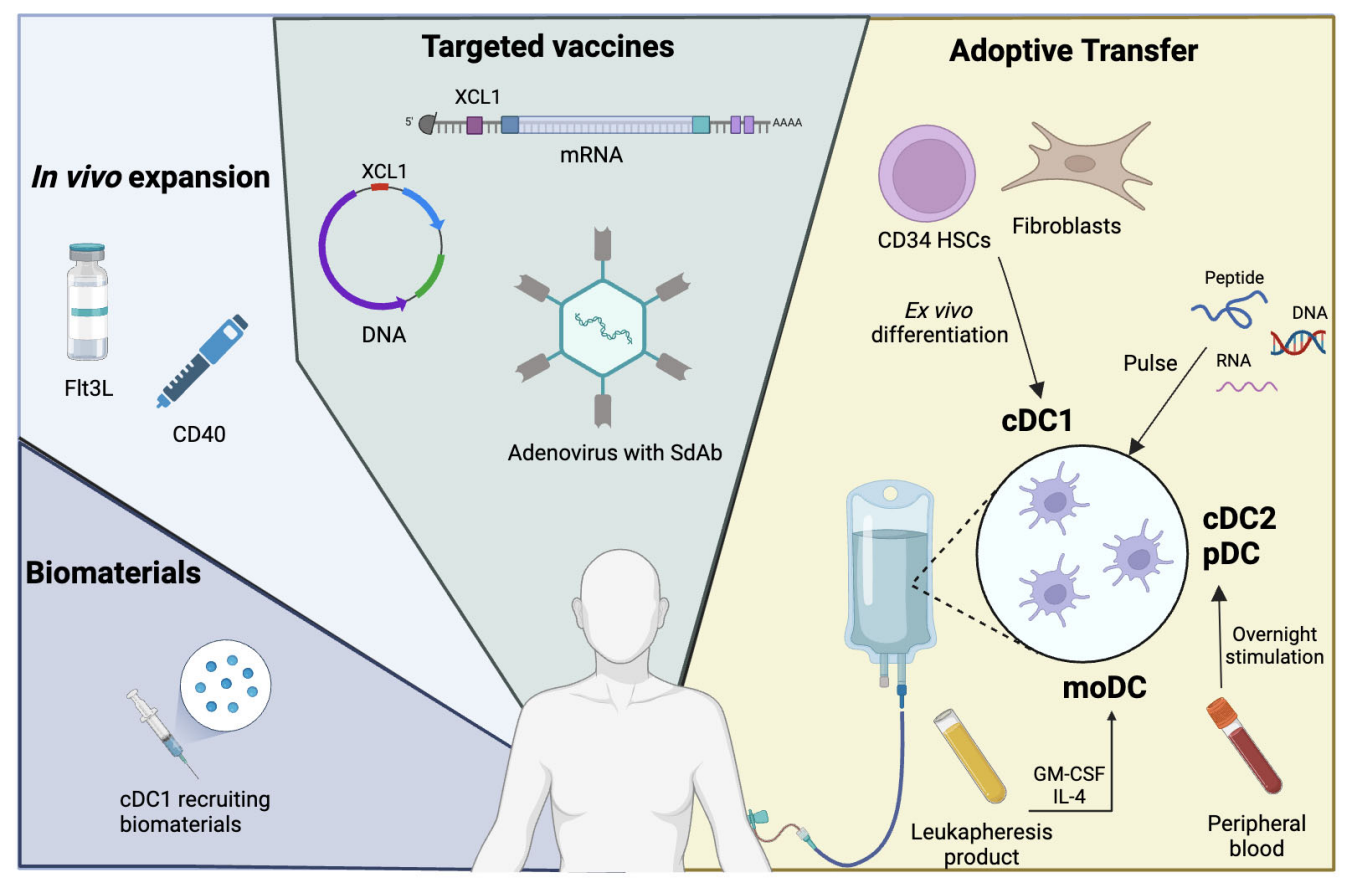
DCs for cancer immunotherapy. DCs are critical to the generation of CD8 T cell anti-tumor activity and may thus be harnessed for therapy. Panel descriptions from right to left: Adoptive Transfer - Given their rarity, DCs may be adoptively transferred after *ex vivo* generation. MoDCs and cDC2s or pDCs may be generated from leukapheresis product or peripheral blood, respectively. cDC1s can be derived *ex vivo* from CD34 HSCs or fibroblasts (iDCs) to sufficient quantities. *Ex vivo* derived DCs may be pulsed with tumor antigen peptides, DNA, or RNA. Targeted Vaccines - Cancer vaccines using mRNA, DNA, or adenoviral platforms may be targeted towards cDC1s by encoding targeting sequences such as XCL1 or by modifying surface proteins in the adenovirus by attachment of antibodies, e.g. single domain antibodies (sdAb). *In vivo* expansion - The quantity of DCs and their activation status in patients may be enhanced through administration of Flt3L or CD40. Biomaterials - Biomaterials, such as injectable mesoporous silica rods (MSRs), may be used to recruit cDC1s to an area of interest, such as site of vaccination.

Although the above summary is not exhaustive, it does shed light on the many receptors, axes, and pathways that are potential targets for cancer immune therapy. Given that DC biology, including subtypes, activation states, and cell-cell interactions, is convoluted, any immune therapy targeting DCs will need to be context-informed. The following sections discuss the past, current, and possible future DC-based immune therapies (**Figure 2**) with a prospective lens, taking what we have learned toward the future—even while acknowledging that the current understanding of DCs is imperfect.

## Role of DCs in existing cancer therapies

### Chemotherapy and radiation therapy enhance tumor immunity that is DC dependent

**Figure 3:**
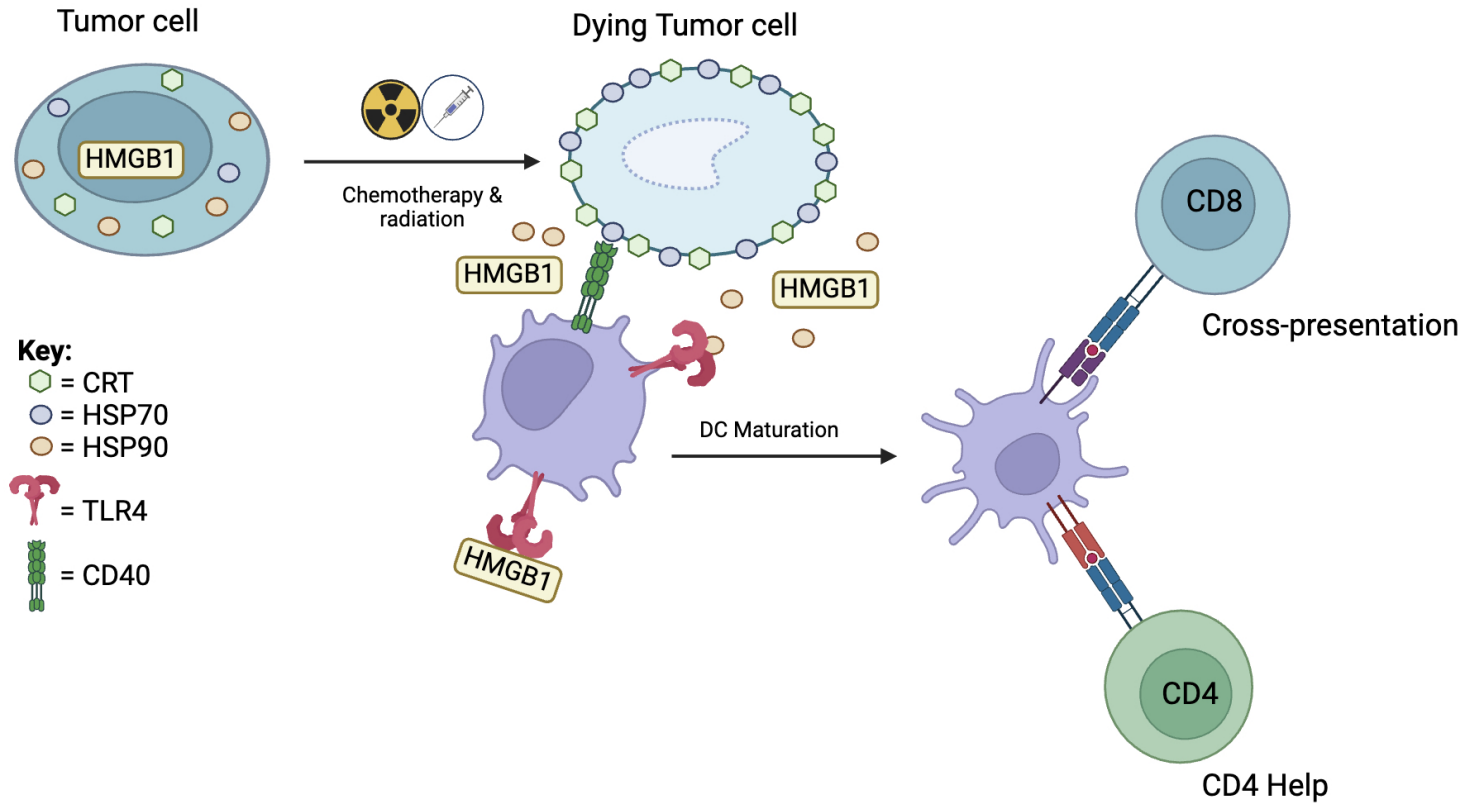
Chemotherapy and radiation therapy induces dendritic cell mediated immunogenic cell death. Immunogenic cell death starts with translocation of calreticulin (CRT) to the cell surface which attracts DCs. This is followed by migration of HSP70 to the cell surface and release of HSP90 and HMGB1. HSP70 interacts with CD40 to induce DC maturation. HSP90 and HMGB1 binding to TLR4 activates downstream pro-inflammatory pathways leading to cross-presentation and recruitment of CD4 help.

Chemotherapy and radiation therapy are important cancer treatment modalities. While these modalities were not designed as immunotherapies, the immune system is critical to their overall effectiveness. Chemotherapy and radiation therapy generate tumor immunity predominantly through induction of DC mediated immunogenic cell death (ICD) of tumor cells (97, 98) (**Figure 3**). After insult by cytotoxic agents, DAMPs such as calreticulin (CRT), HSP70 and HSP90, HMGB1, and ATP are released. Within 1 hour, CRT translocates from the ER to the cell surface, acting as an “eat me” signal and binding to CD91 on the DC cell membrane. CRT-CD91 interaction is necessary for antigen cross-presentation to CTLs (99). CRT expression has been found to correlate with clinical outcome in non-small cell lung cancer and is associated with higher tumor infiltration of mDCs and effector memory T cell subsets (100).

Twelve hours after insult, molecular chaperones HSP70 and HSP90 appear on the cell surface of dying cells. HSP70 interacts with CD40 on DCs, causing upregulation of CD86 and further increase in CD40 expression, which are co-stimulatory signals for CD8 T cells (101, 102). Furthermore, HSP70 stimulates TLR4 on DCs and activates downstream pro-inflammatory pathways (103). HSP90 binding with CD91 facilitates cross-presentation and leads to DC activation and upregulation of CD80, CD83, and CD86 which are associated with DC maturation (104). In late-stage ICD, necrotic cells release HMGB1 and ATP. Binding of extracellular HMGB1 to TLR4 induces efficient cross-presentation by DCs (105). Binding of ATP to P2X7 receptor on DCs leads to secretion of IL-1β necessary for promoting IFN-γ producing CD8 T cells.

### Checkpoint inhibitors and DCs

Immune checkpoint therapy (ICT) is one of the great success stories of modern cancer immunotherapy. The greatest successes have been achieved targeting PD-1/PD-L1 and CTLA-4. Tumor cells escape cancer immunosurveillance through activation of immune checkpoint pathways (106). Monoclonal antibodies and molecules interrupting these inhibitory pathways help to reinvigorate tumor immunity and has translated to clinical success in numerous solid and hematologic malignancies (107). While the focus of immune checkpoint inhibitors has been on T cells, many of the checkpoint receptors have ligands on antigen presenting cells including DCs. While there are numerous immune checkpoint receptors and ligands, we will focus our discussion on the PD-L1-PD1 and CTLA-4 pathways which have FDA approved therapies.

Programmed death-1 (PD-1) is a hallmark of T cell exhaustion, a dysfunctional phenotype arising from chronic antigenic and inflammatory stimulation. While PD-L1 signaling arises predominantly from tumor-associated macrophages, PD-L1 on DCs is an important regulator of tumor immunity, and PD-L1-PD-1 signaling restricts T-cell responses during cross-presentation (108). Intravital real-time imaging with single cell RNA sequencing analysis demonstrates that the full tumor effect of anti-PD-1 requires T cell and DC crosstalk and DC-derived IL-12 (65). Furthermore, neoadjuvant anti-PD-1 therapy has been shown to induce both T cell and cDC1 activation in glioblastoma (109).

Cytotoxic T-lymphocyte-associated protein-4 (CTLA-4) is another crucial negative regulator of T-cell function. Mature DCs have been shown to express high intracellular levels of CTLA-4; secretion of CTLA-4 via microvesicles downregulates CD80/CD86 on bystander DC through competitive binding, resulting in downstream inhibitory implications for T cell function (110). It has also been observed that binding of CTLA-4 on MoDCs by agonist antibodies enhances IL-10 secretion, thus decreasing T cell proliferation (111).

## DC vaccines (adoptive transfer)

The last three decades have seen a precipitous increase in the number of clinical trials featuring DC vaccines. The majority are autologous and monocyte-derived, with some variation in subtype in recent years as outlined below. In general, tumor antigens are introduced to patient DCs *ex vivo* prior to adoptive transfer. With the trend toward personalized vaccines, tumor neoantigens are increasingly used (**Table 1**).

**Table 1:** Neoantigen DC vaccines undergoing clinical trial.

NCT Number	Conditions	DC type	Antigen Delivery	Arms	Phase	Study Status	Estimated Completion Date	Results
**NCT02632019**	Advanced biliary tract malignancy			Arm 1: Gemcitabine + Dendritic cell-precision T cell for neo-antigen (DC-PNAT) Arm 2: Gemcitabine	1, 2	Unknown	2017–03	
**NCT04879888**	Triple negative breast cancer		Peptide	DC Vaccine	1	Complete	2017–07	Case report on one patient: Positive antigen-specific T cell response (112)
**NCT03205930**	NSCLC Stage IV		Peptide (TAA + neoantigen)	Neoantigen Multiple Target Antigen Stimulating Cell Therapy (Neo-MASCT)	1, 2	Unknown	2019–11	
**NCT02956551**	NSCLC	moDC	Peptide or oxidized tumor lysate	DC vaccine	1	Complete	2020–06	Positive neoantigen-specific T cell response in 10/10 patients; 3/12 achieved PR; Median PFS 5.5 months, Median OS 7.9 months at median follow-up 7.1 months (113)
**NCT03674073**	HCC			Arm 1: DC Vaccine + Microwave Ablation Arm 2: Microwave Ablation	1	Unknown	2020–12	
**NCT03871205**	NSCLC, SCLC			DC Vaccine	1	Unknown	2020–12	
**NCT04105582**	Triple Negative Breast Cancer		Peptide	DC Vaccine	1	Complete	2022–01	
**NCT03914768**	Diffuse Intrinsic Pontine Glioma or Glioblastoma		Whole tumor or peptide	DC Vaccine	1	Unknown	2022–12	
**NCT03067493**	Primary HCC		Peptide (TAA + neoantigen)	Arm 1: Neoantigen Multiple Target Antigen Stimulating Cell Therapy (Neo-MASCT) Arm 2: Observation	2	Recruiting	2023–03	Interim report: Positive neoantigen-specific immune response in 6/7 patients; no clinical outcomes data (114)
**NCT05235607**	Melanoma, Bladder cancer, Colorectal cancer			DC vaccine + Autologous sensitized T cells	1	Unknown	2023–12	
**NCT05270720**	Recurrent Ovarian Cancer	moDC	mRNA	DC Vaccine	1	Unknown	2024–03	
**NCT05317325**	Esophageal Squamous Cell Carcinoma		Peptide or oxidized tumor lysate	OCDC vaccine (oxidized tumor lysate - prime) followed by NeoDC vaccine (neoantigen peptide - boost)	1	Unknown	2024–04	
**NCT05195619**	Metastatic NSCLC	moDC	Peptide	Arm 1: Patients w/o actionable driver mutations: PEP-DC vaccine + Low-dose cyclophosphamide Arm 2: Patients with actionable driver mutations: PEP-DC vaccine + Low-dose cyclophosphamide	1	Recruiting	2024–09	
**NCT04968366**	Glioblastoma		Peptide	DC Vaccine +Temozolomide	1	Active, not recruiting	2024–12	
**NCT01885702**	Colorectal Cancer with MSI, Lynch Syndrome	moDC	Peptide	DC Vaccine	1, 2	Active, not recruiting	2024–12	
**NCT06253234**	WHO Grade 3 or 4 Gliomas		Peptide	ZSNeo-DC1.1	1	Recruiting	2025–02	
**NCT04912765**	HCC, Colorectal cancer			DC vaccine + Nivolumab	2	Recruiting	2025–05	
**NCT04078269**	NSCLC	moDC	mRNA	DC Vaccine	1	Active, not recruiting	2025–12	Positive T cell response in 5/6 patients; 3/6 patients with disease recurrence at 2 year follow-up (115)
**NCT05749627**	Primary or metastatic melanoma, Gastrointestinal tumor, Breast cancer, Cervical cancer, Pancreatic cancer, Lung cancer, or other malignant tumors			Arm 1: DC Vaccine Arm 2: Peptide Vaccine	Not applicable	Recruiting	2025–12	
**NCT05767684**	Stage IV pancreatic cancer, liver cancer, biliary tract cancer and colorectal cancer			Arm 1: DC Vaccine Arm 2: DC Vaccine + Lenvatinib + Nivolumab	1	Recruiting	2026–03	
**NCT06195618**	Triple negative breast cancer		Peptide	DC Vaccine	1	Not yet recruiting	2026–06	
**NCT04147078**	Gastric Cancer| Hepatocellular Carcinoma| Non-Small-Cell Lung Cancer| Colon Rectal Cancer		Peptide	DC Vaccine	1	Recruiting	2026–06	Interim report: 5/13 patients generated a positive immune response to at least 50% of neoantigens, median survival outcome not reached (116)
**NCT06329908**	Advanced lung cancer resistant to ICI		Peptide (TAA + neoantigen)	Neo-DCVac/LG002 + PD1/PDL-1 Inhibitors	1	Recruiting	2026–10	
**NCT05886439**	Advanced Lung Carcinoma		mRNA	Arm 1: NSCLC DC Vaccine (LK101) + Pembrolizumab Arm 2: SLCLC DC Vaccine (LK101) + Durvalumab	1	Recruiting	2026–12	
**NCT05631886**	Lymphoma with EphA2 Overexpression and TP53 R273H/R175H/R248Q/R249S mutation	CAR-DC	Peptide (Tp53)	Arm 1: TP53-EphA-2-CAR-D + Abraxane + Cyclophosphamide Arm 2: Arm 1+ Anti-PD-1 Antibody Arm 3: Arm 2 + Anti-CTLA4	1	Recruiting	2026–12	
**NCT05631899**	Solid Tumors with EphA2 Overexpression and KRAS G12V/G12C/G12D mutation	CAR-DC	Peptide (KRAS)	Arm 1: KRAS-EphA-2-CAR-DC + Abraxane + Cyclophosphamide Arm 2: Arm 1+ Anti-PD-1 Antibody Arm 3: Arm 2 + Anti-CTLA4	1	Recruiting	2026–12	
**NCT06342908**	Diffuse Hemispheric Glioma, H3 G34-Mutant		Peptide	DC Vaccine + PolyIC-LC	1	Not yet recruiting	2028–01	
**NCT04627246**	Pancreatic Adenocarcinoma		Peptide	PEP-DC + Nivolumab	1	Recruiting	2028–09	
**NCT05714306**	High Grade Serous Ovarian Carcinoma	moDC	Peptide	Arm 1: PEP-DC1 (peptide) + Low-dose cyclophosphamide Arm 2: OC-DC (oxidized tumor lysate) + Low-dose cyclophosphamide followed by PEP-DC2 (peptide) + Low-dose cyclophosphamide	1, 2	Not yet recruiting	2030–03	

Blank cells represent unknown data that has not been reported.

NSCLC, Non-small cell lung cancer; HCC, Hepatocellular cancer; ICI, Immune checkpoint inhibition; TAA, Tumor associated antigen; PR, Partial response; PFS, Progression free survival; OS, Overall survival.

### Monocyte-derived (moDC)

In 2010, Sipuleucel-T (Provenge) became the first and to date only dendritic cell therapy approved by the U.S. Food and Drug Administration (117). It was authorized for the treatment of asymptomatic metastatic castration-resistant prostate cancer. The therapy involves treating patient-derived moDCs with a chimeric protein of PAP (a prostate specific antigen) and GM-CSF (118). In the phase III IMPACT trial of the therapy, an improvement in median survival of 4.1 months (21.7 vs. 25.8 months) was observed in the treatment group compared to standard therapy, although there was no difference in time to progression by PSA levels (119). The performance of Sipleucel-T as a monotherapy ultimately was never widely adopted due to high cost for limited benefit. It is however currently being investigated for use as combination therapy with checkpoint blockade, chemotherapy, radiation, and cryoablation (120, 121). Again, clinical benefit has been unclear. In the VIABLE trial, despite promising immune efficacy demonstrated in phase I/II trials, autologous moDC vaccination in combination with docetaxel had no survival or progression-free survival in metastatic prostate cancer patients upon phase III evaluation (122).

In the more than a decade since Sipuleucel-T, numerous clinical trials of cancer DC therapies of various forms have been registered. These include *ex vivo* generated DCs transfected with mRNA (123) or pulsed with peptides encoding tumor antigens (124), DCs fused with patient derived tumor cells (125), or tumor cells modified to secrete DC stimulating cytokines (GVAX) (126). While most trials demonstrate an overall survival benefit, therapies often fail to demonstrate a clear tumor response, leaving room for therapeutic optimization (127). For example, moDCs have shown success when combined with other therapeutic modalities such as immune checkpoint blockade. In a phase II advanced stage melanoma trial, mRNA electroporated moDCs plus ipilimumab had a 38% tumor response rate with 8 out of 15 responders demonstrating response beyond a median of 36 months (128). Furthermore, moDC vaccinations may be more effective in the adjuvant setting after surgery with minimal residual tumor rather than in the setting of metastatic disease (129, 130).

A key limitation to these therapies may be the predominance of moDCs instead of other types of DCs. The choice of moDCs is one of convenience since they are generated with relative ease through culturing patient bone marrow or blood-derived myeloid cells with GM-CSF and IL-4. As mentioned previously, however, compared to “natural” conventional DCs in particular, moDCs are innately less potent in antigen cross-presentation and induction of CD8 T cell responses (58). Furthermore, they lack migration potential, which may mitigate their ability to induce *in vivo* immune responses (131). To address these shortcomings, one study introduced a monocyte-derived DC culture protocol that used TNF-α, IL-1b, Poly-ICLC, IFN-α, and IFN-γ to mature “alpha-type-1-polarized-DC” (alphaDC1) that exhibit superior migratory potential and secrete high levels of IL-12 (132), although reproducibility and scalability of this method has been called into question (133). In-human trial data is limited and in disparate settings, with alphaDC1 showing some antitumor efficacy in a few recurrent malignant glioma patients but no objective clinical efficacy in patients with malignant peritoneal disease (134, 135). Of note, both these trials utilized intranodal alphaDC1 vaccination, whereas an ongoing trial NCT05127824 in non-metastatic clear cell renal cell carcinoma utilizes intradermal injection, which will be a test of the purported increased migratory capacity of alphaDC1.

### cDC2 and plasmacytoid DC vaccines

In response to inconsistent clinical efficacy, requirement of extensive *ex vivo* culture periods, and relatively inefficient antitumor T cell induction, the next era of dendritic cell vaccines has moved toward cDC2 and plasmacytoid DC vaccines. In the last decade, several trials have evaluated autologous pDC and cDC2 vaccines in metastatic melanoma patients, as well as in combination (both pDC and cDC2 vaccination) for stage 3 melanoma and prostate cancer (136). Individually and together, pDC and cDC2 are able to induce antigen-specific T cell responses in phase II trials (136–139). However, interim results from a subsequent phase III trial (NCT02993315) evaluating combination pDC and cDC2 therapy in stage 3 melanoma demonstrated no improvement in 2-year RFS over placebo (140). A considerable benefit of these vaccines was that pDC and cDC2 isolated from the patient’s peripheral blood only needed to be cultured overnight, enabling high-throughput production. However, as long-term clinical efficacy remains to be seen, there is apparent room for optimization. A proposed mechanism impeding full efficacy for cDC2 vaccines is the presence of soluble factors in the TME such as IL-6 and M-CSF that convert cDC2s to a tolerogenic state upon arrival (141). One group has taken an antigen-agnostic approach that bypasses both *ex vivo* culture and need for tumor infiltration by injecting unpulsed cDC2 intratumorally. This method has been attempted in two phase 1 trials of advanced melanoma (NCT03747744) and various advanced solid tumors (NCT03707808) with the most durable tumor response seen in melanoma (142, 143). An obvious challenge to intratumoral injection is feasibility in visceral tumors, and, despite partial and even complete tumor response seen in some melanoma patients, the majority of patients still progressed (143).

### The potential for cDC1 vaccines

Despite overwhelming acknowledgement of the superiority of cDC1 for priming CTLs, cDC1 vaccine therapy remains nascent. The phase 1 trial (NCT03747744) highlighted above utilizing intratumoral DC injections also evaluated a combined cDC2 and cDC1 arm. One out of six patients experienced a partial tumor response. Notably, after leukapheresis, the cDC1 yield was ten-fold lower on average than the cDC2 yield, and purity was merely 5.8% (143). Herein lies the challenge, for relative scarcity of cDC1 has precluded in-human utility of a cDC1 vaccine. Yet, recognizing the potential for cDC1-mediated antitumor immunity, recent efforts toward *ex vivo* generation remain undeterred (**Table 2**).

**Table 2:** Strategies to generate cDC1s *ex vivo*.

Authors	Input	Output	Expansion	Differentiation
**Poulin et al.**	Lin- cord blood	CD141+/Clec9a+ cDC	***7–11 days*** Flt3L 100 ng/ml SCF 100 ng/ml IL-3 20 ng/ml IL-6 20 ng/ml	***12–14 days*** Flt3L 100 ng/ml SCF 20 ng/ml GM-CSF 20 ng/ml IL-4 20 ng/ml
**Balan et al.**	Cord blood CD34+	XCR1+ cDC	***7 days*** Flt3L 100 ng/ml SCF 100 ng/ml IL-3 20 ng/ml IL-6 20 ng/ml	***8–11 days*** Flt3L 100 ng/ml SCF 20 ng/ml GM-CSF 20 ng/ml IL-4 20 ng/ml
**Balan et al.**	Cord blood CD34+	XCR1+ cDC	***7 days*** Flt3L 100 ng/ml SCF 100 ng/ml IL-3 20 ng/ml TPO 50 ng/ml	***8–13 days*** Flt3L 100 ng/ml SCF 20 ng/ml GM-CSF 20 ng/ml IL-4 20 ng/ml
**Helft et al.**	MLP or CMP	Clec9a+ cDC	***12 days*** Flt3L 100 ng/ml SCF 20 ng/ml GM-CSF 20 ng/ml IL-4 20 ng/ml	
**Lee et al.**	Cord blood CD34+	CD141+ cDC	***14 days*** MS5 stromal cells Flt3L 100 ng/ml SCF 20 ng/ml GM-CSF 10 ng/ml	
**Kirkling et al.**	Bone marrow CD34+	Clec9a+ cDC	***14 days*** OP9-DL1 or OP9-DL4 cells Flt3L 100 ng/ml SCF 20ng/ml GM-CSF 20 ng/ml	

Protocols have been developed to generate CD141+/CLEC9A+ DCs from CD34+ progenitor cells isolated from human cord blood or bone marrow. These are permissive cultures featuring cytokines such as FLT3L and SCF in combination with GM-CSF, IL-3, IL-4, IL-6, TPO, and other cytokines. There are typically 2 phases in such protocols, including an “expansion” phase, to expand pluripotent progenitors, and a “differentiation” phase to allow differentiation of those progenitors into various lineages of dendritic cells, including cDC1s (144–148). *Ex vivo* generated cDC1s resemble natural cDC1s in their gene expression profile and in their phenotype by their response to TLR3 signaling by PolyIC and increased IL-12 production after T cell stimulation through CD40, IL-4, GM-CSF, or IFN-γ (144). Although these strategies are successful in generating cells that resemble cDC1s in genotype and phenotype, quantity remains a barrier. Lee et al. reports that per thousand CD34+ progenitor input, *ex vivo* differentiation yields a mere 0.2–0.3 CD141+ cDCs (147).

Another approach to generate cDC1s is instructive, rather than permissive. Rosa et al. demonstrated that fibroblasts could be reprogrammed into induced DCs (iDCs) that behave like cDC1s. They found for human and mouse fibroblasts, transcription factors PU.1, IRF8, and BATF3 transfected via lentiviral vectors were sufficient for reprogramming. These iDCs functionally have increased CD40 and CD86 expression and secrete IL-12 after TLR stimulation like native cDC1s and also demonstrate similar single-cell transcriptomes. As in cytokine culture, quantity remains a barrier for translation, with only 0.6% and 0.2% of human embryonic and dermal fibroblasts reprogrammed respectively (149).

## DC activation and expansion *in vivo*

Whereas DC vaccines often rely on *ex vivo* manipulation, other DC-based strategies strive to leverage agents that expand or activate DCs *in vivo*. Below are two key therapies that have been recently explored.

### FLT3L expansion

FLT3/FLT3L is an essential pathway in the early development of dendritic cells. FLT3L administration resulted in strong expansion of cDC1s in mice (150). This was associated with increased proliferation of tumor specific CD8 T cells in tumor draining lymph nodes (151). Of note, FLT3L administration has been shown to inhibit tumor growth in fibrosarcoma (152), breast (153), melanoma, and lymphoma cancer models (154). Hegde et al. demonstrated that PDACs have a paucity of cDCs and increasing cDC number through FLT3L treatment restored immune responses to cancer antigens, inhibited tumor growth, reversed fibrosis, and sensitized tumors to radiation therapy (69).

FLT3L therapy has been tested in multiple clinical trials in the last two decades. FLT3L as a monotherapy tends to increase circulating DCs without objective clinical response (155, 156). As an adjuvant to vaccine, it has mixed effects. With a HER-2/neu peptide vaccine, FLT3L administration for 14 days was not able to elicit any T cell proliferation, but it may have led to an increase in frequency of precursor IFN-γ+ HER2-specific T cells (157). However, with a DC-targeted anti-DEC205-NY-ESO-1 vaccine, FLT3L administration was associated with activation and proliferation of circulating effector T cells as well as NK cells and DCs (158). Most recently, a combination of adenoviral vectors Ad-hCMV-TK “Ad-TK” and Ad-hCMV-FLT3L used to treat glioma patients was able to induce a significant influx of intratumoral CD8 T cells and pDCs, a finding not seen in prior studies using only Ad-TK (159). Regarding toxicity, while generally found to be tolerable in small safety studies, FLT3L has been associated with adverse effects ranging from local inflammation of the skin to lymphoproliferative disorders in severe cases (160). FLT3L therefore remains a promising therapy as an adjuvant, but will need close attention to tolerability in larger studies.

### CD40 activation

CD40 agonism is an attractive way to enhance and/or replace T cell help and drive both CD8 T cell responses (8, 161) and T-cell mediated tumor immunity (162–164). While CD40 agonism broadly activates a variety of hematopoietic cell types that include B cells and macrophages, its activation of DCs, in particular cDC1s, plays an essential role in tumor immunity as illustrated by abrogation of tumor control in murine *Batf3^-/-^* models (162, 165). In a small percentage of malignancies, direct cytotoxicity from CD40 ligation on tumor cells (166, 167) creates a “vaccine effect” in which tumor antigens are released and subsequently acquired and cross-presented by intratumoral APCs. On the other hand, in tumors that feature low CD40 expression, fail to express strong antigens, or are characterized by poor immune infiltrate, CD40 agonist therapy has shown promise as an adjunct to multiple cancer therapies, including radiation, irreversible electroporation, neoantigen vaccine, chemotherapy, FLT3L, and checkpoint blockade (69, 162, 168–171). Furthermore, in multiple anti-PDL-1 resistant murine tumors with poor T-cell infiltrate, a combination of FLT3L, radiation, polyIC, and CD40 agonist was able to reverse anti-PDL-1 resistance, induce tumor regression, and establish antitumor memory (169).

With the success in preclinical models, there are now multiple clinical trials testing CD40 agonism as monotherapy or in combination with other cancer therapies. Current trials with immune therapy take advantage of CD40 agonism in different ways, such as an adenoviral vector vaccine encoding TAA MUC1 and CD40-ligand in multiple advanced adenocarcinomas (172), or TriMixDC-Mel-IPI, a monocyte-derived dendritic cell encoding multiple melanoma TAAs as well as CD70, CD40 ligand, and TLR4 (173). The more traditional approach to CD40 agonism is by administering CD40 agonistic antibodies, of which there are several different variations (174). In a phase II trial of metastatic melanoma patients who had progressed on checkpoint blockade, the combination of sotigalimumab (APX005M CD40 agonist antibody) and nivolumab was able to induce long-lasting clinical tumor response in a subset of patients (175). On the contrary, in the PRINCE II trial, sotigalimumab with gemcitabine/nab-paclitaxel and/or nivolumab in metastatic pancreas cancer showed little clinical benefit. In this trial, those who did have some survival benefit in the sotigalimumab/chemotherapy arm were shown to have higher pre-treatment frequencies of cross-presenting dendritic cells and Tbet+ CD4 Th1 cells, indicating possible utility for biomarker use in treating these patients with similar therapy (176). Of note, agonistic antibodies are reported in several trials to have significant systemic side effects, including cytokine release syndrome and hepatotoxicity (174); the next generation of CD40 agonistic antibodies will need to bypass systemic toxicity, with some methods already being explored including local administration or engineered tumor-specific antibodies (177).

A potent activator of dendritic cells, CD40 agonism is a reasonable adjunct to multiple cancer therapies. Its antitumor activity is clear in a multitude of preclinical studies and it has been shown to produce modest clinical benefit in certain patient populations. In a strongly immunosuppressive TME, CD40 agonism will be more likely to succeed with a cytotoxic agent (i.e. chemotherapy or radiation), an expansion/infiltration agent (i.e. FLT3L), and/or tolerogenic reversal (i.e. checkpoint blockade).

## DC targeting

Cancer vaccines using tumor associated antigens and neoantigens have risen to prominence, utilizing an array of vaccine platforms such as DNA, mRNA, peptides, and viral vectors with many ongoing clinical trials (clinicaltrials.gov). Given the variety of DCs, heterogeneity among subsets, and context-dependent activation states even within each subset, strategies to target tumor antigens to DCs of interest, particularly cDC1, are gaining popularity. Especially with the challenges of generating enough cDC1s *ex vivo*, targeting and activating cDC1s *in vivo* is an attractive strategy with great translational potential. Vaccines encoding tumor antigens targeted towards cDC1s in mice have shown increased T cell responses against the encoded antigens. Most targeting strategies rely on the unique surface markers of cDC1s for targeting such as XCR1 and CLEC9A (178–180). For instance, adenovirus vaccine platforms may be modified to replace its native fiber with CD40L (181) or single domain antibodies for CLEC9A or XCR1 to specifically infect cDC1s (182, 183). Furthermore, DNA and RNA vaccines may encode XCL1 chemokine so its protein products will be delivered to XCR1+ DCs (41, 184). Similarly, peptide vaccination of OVA synthetic long peptide (SLP) fused with XCL1 led to greater tumor control of B16-OVA melanoma tumors with higher CD8 T cell tumor infiltration (185).

Biomaterials may be used to enhance DC targeting. An alternative to targeting natural receptors on DCs is to introduce artificial targets. This has the advantage of producing DCs with a significantly greater density of chemical tags available for targeting (186). An example of such a strategy is the use of unnatural sugars. These sugars are endocytosed and metabolized by DCs and byproducts are subsequently presented as unique glycoproteins and glycolipids on the cell membrane (187). T cells, proteins, and cytokines can subsequently be targeted towards these unique chemical tags. Biomaterials may also be used as scaffolds to recruit DCs to areas of interest. Mesoporous silica rods (MSRs), for example, that self-assemble upon subcutaneous injection carrying immunomodulatory agents have been shown to increase efficacy of vaccines through recruitment of conventional dendritic cells in mice (188, 189). Recruitment of DCs via a stabilized XCL1 has shown similar results (178).

## Conclusion

Being key mediators of CD8 T cell responses, dendritic cells have tremendous potential for cancer immunotherapy. To date however, the performance of DC-based therapies has been limited. Improved understanding of the immunobiology of dendritic cells reveals a heterogeneous group of cells with subsets having unique functions (38). Previous therapies have focused on using moDCs given their relative abundance. While capable of cross-presenting antigens *in vitro*, they possess restricted migration ability and are less potent at priming and activating CD8 T cells compared to other DC subsets, limiting their effectiveness *in vivo* (56, 58). Alternatively, autologous pDC and cDC2 therapies are a feasible approach to DC vaccination with quick turnaround time after leukapheresis and isolation (136), but each subset is poorly understood and can be driven into protumor states, which may preclude clinical efficacy.

Among natural DCs, cDC1s have been identified as the key mediators of CTL immunity because they effectively cross-present tumor antigens. Pre-clinical studies suggest the utilization of cDC1s for cancer immunotherapy may be much more rewarding compared to the previous generation of moDC therapies. However, one fundamental roadblock to effective utilization of cDC1s is generating sufficient quantities of cells. A currently ongoing phase I/II trial in stage III ovarian cancer (NCT05773859) may give us further insight into feasibility. Meanwhile, this challenge may be overcome through various strategies such as *in vivo* expansion or *in vivo* targeting, as delineated above.

The efficacy of any DC therapy will likely need to be through a highly tailored approach to both patient and disease. For example, DC vaccination is suggested to best benefit patients with both low tumor burden and low tumor mutational burden (190). Additionally, there is growing reliance on tumor-specific biomarkers to predict not only response to DC therapy but choice of adjunct therapy, whether it be chemotherapy, radiation, and/or immune therapy (191). Ultimately, understanding the intrinsic nuances of the TME is crucial to identifying barriers to immune activation and will dictate target DC subset and phenotype, as well as therapeutic platform, route of administration, timing, and synergy of combinatorial treatment.

## Author contributions

MC: Writing – original draft, Writing – review & editing. FZ: Writing – original draft, Writing – review & editing. SG: Writing – review & editing. WG: Writing – review & editing.

## References

[1] Sharma P Allison JP. Dissecting the mechanisms of immune checkpoint therapy. Nat Rev Immunol. (2020) 20:75–6. doi: 10.1038/s41577-020-0275-8 31925406

[2] Kershaw MH Westwood JA Darcy PK. Gene-engineered T cells for cancer therapy. Nat Rev Cancer. (2013) 13:525–41. doi: 10.1038/nrc3565 23880905

[3] Li L Goedegebuure S Gillanders WE. Preclinical and clinical development of neoantigen vaccines. Ann Oncol. (2017) 28:xii11–xii7. doi: 10.1093/annonc/mdx681 PMC5834106 29253113

[4] Chen Daniel S Mellman I. Oncology meets immunology: the cancer-immunity cycle. Immunity. (2013) 39:1–10. doi: 10.1016/j.immuni.2013.07.012 23890059

[5] Wu R Murphy KM. DCs at the center of help: Origins and evolution of the three-cell-type hypothesis. J Exp Med. (2022) 219:e20211519. doi: 10.1084/jem.20211519 35543702 PMC9098650

[6] Borst J Ahrends T Bąbała N Melief CJ Kastenmüller W. CD4+ T cell help in cancer immunology and immunotherapy. Nat Rev Immunol. (2018) 18:635–47. doi: 10.1038/s41577-018-0044-0 30057419

[7] Laidlaw BJ Craft JE Kaech SM. The multifaceted role of CD4+ T cells in CD8+ T cell memory. Nat Rev Immunol. (2016) 16:102–11. doi: 10.1038/nri.2015.10 PMC4860014 26781939

[8] Ridge JP Di Rosa F Matzinger P. A conditioned dendritic cell can be a temporal bridge between a CD4+ T-helper and a T-killer cell. Nature. (1998) 393:474–8. doi: 10.1038/30989 9624003

[9] Melssen M Slingluff CL Jr. Vaccines targeting helper T cells for cancer immunotherapy. Curr Opin Immunol. (2017) 47:85–92. doi: 10.1016/j.coi.2017.07.004 28755541 PMC5757837

[10] Ferris ST Ohara RA Ou F Wu R Huang X Kim S. cDC1 vaccines drive tumor rejection by direct presentation independently of host cDC1. Cancer Immunol Res. (2022) 10:920–31. doi: 10.1158/2326-6066.c.6550934 PMC9357132 35648641

[11] Gardner A de Mingo Pulido A Ruffell B. Dendritic cells and their role in immunotherapy. Front Immunol. (2020) 11:924. doi: 10.3389/fimmu.2020.00924 32508825 PMC7253577

[12] Norian LA Rodriguez PC O’Mara LA Zabaleta J Ochoa AC Cella M. Tumor-infiltrating regulatory dendritic cells inhibit CD8+ T cell function via L-arginine metabolism. Cancer Res. (2009) 69:3086–94. doi: 10.1158/0008-5472.CAN-08-2826 PMC2848068 19293186

[13] Terra M Oberkampf M Fayolle C Rosenbaum P Guillerey C Dadaglio G. Tumor-derived TGFβ Alters the ability of plasmacytoid dendritic cells to respond to innate immune signalingTGFβ Produced by tumor cells alters the functions of pDCs. Cancer Res. (2018) 78:3014–26. doi: 10.1158/0008-5472.CAN-17-2719 29523540

[14] Plesca I Müller L Böttcher JP Medyouf H Wehner R Schmitz M. Tumor-associated human dendritic cell subsets: Phenotype, functional orientation, and clinical relevance. Eur J Immunol. (2022) 52:1750–8. doi: 10.1002/eji.202149487 35106759

[15] Tesfaye DY Gudjonsson A Bogen B Fossum E. Targeting conventional dendritic cells to fine-tune antibody responses. Front Immunol. (2019) 10. doi: 10.3389/fimmu.2019.01529 PMC6620736 31333661

[16] Theisen DJ Davidson JT IV Briseño CG Gargaro M Lauron EJ Wang Q. WDFY4 is required for cross-presentation in response to viral and tumor antigens. Science. (2018) 362:694–9. doi: 10.1126/science.aat5030 PMC6655551 30409884

[17] Wakim LM Bevan MJ. Cross-dressed dendritic cells drive memory CD8+ T-cell activation after viral infection. Nature. (2011) 471:629–32. doi: 10.1038/nature09863 PMC3423191 21455179

[18] Das Mohapatra A Tirrell I Benechet AP Pattnayak S Khanna KM Srivastava PK. Cross-dressing of CD8alpha(+) dendritic cells with antigens from live mouse tumor cells is a major mechanism of cross-priming. Cancer Immunol Res. (2020) 8:1287–99. doi: 10.1158/2326-6066.CIR-20-0248 32759362

[19] MacNabb BW Tumuluru S Chen X Godfrey J Kasal DN Yu J. Dendritic cells can prime anti-tumor CD8(+) T cell responses through major histocompatibility complex cross-dressing. Immunity. (2022) 55:982–97 e8. doi: 10.1016/j.immuni.2022.04.016 35617964 PMC9883788

[20] Dolan BP Gibbs KD Jr. Ostrand-Rosenberg S. Dendritic cells cross-dressed with peptide MHC class I complexes prime CD8+ T cells. J Immunol. (2006) 177:6018–24. doi: 10.4049/jimmunol.177.9.6018 17056526

[21] Squadrito ML Cianciaruso C Hansen SK De Palma M. EVIR: chimeric receptors that enhance dendritic cell cross-dressing with tumor antigens. Nat Methods. (2018) 15:183–6. doi: 10.1038/nmeth.4579 PMC5833950 29355847

[22] Ruhland MK Roberts EW Cai E Mujal AM Marchuk K Beppler C. Visualizing synaptic transfer of tumor antigens among dendritic cells. Cancer Cell. (2020) 37:786–99.e5. doi: 10.1016/j.ccell.2020.05.002 32516589 PMC7671443

[23] Allan RS Waithman J Bedoui S Jones CM Villadangos JA Zhan Y. Migratory dendritic cells transfer antigen to a lymph node-resident dendritic cell population for efficient CTL priming. Immunity. (2006) 25:153–62. doi: 10.1016/j.immuni.2006.04.017 16860764

[24] Gurevich I Feferman T Milo I Tal O Golani O Drexler I. Active dissemination of cellular antigens by DCs facilitates CD8(+) T-cell priming in lymph nodes. Eur J Immunol. (2017) 47:1802–18. doi: 10.1002/eji.201747042 28872666

[25] Ebrahimi-Nik H Corwin WL Shcheglova T Das Mohapatra A Mandoiu II Srivastava PK. CD11c(+) MHCII(lo) GM-CSF-bone marrow-derived dendritic cells act as antigen donor cells and as antigen presenting cells in neoepitope-elicited tumor immunity against a mouse fibrosarcoma. Cancer Immunol Immunother. (2018) 67:1449–59. doi: 10.1007/s00262-018-2202-4 PMC6132860 30030558

[26] Yewdall AW Drutman SB Jinwala F Bahjat KS Bhardwaj N. CD8+ T cell priming by dendritic cell vaccines requires antigen transfer to endogenous antigen presenting cells. PloS One. (2010) 5:e11144. doi: 10.1371/journal.pone.0011144 20585396 PMC2886840

[27] Schoenberger SP Toes RE van der Voort EI Offringa R Melief CJ. T-cell help for cytotoxic T lymphocytes is mediated by CD40-CD40L interactions. Nature. (1998) 393:480–3. doi: 10.1038/31002 9624005

[28] Ahrends T Spanjaard A Pilzecker B Babala N Bovens A Xiao Y. CD4(+) T cell help confers a cytotoxic T cell effector program including coinhibitory receptor downregulation and increased tissue invasiveness. Immunity. (2017) 47:848–61 e5. doi: 10.1016/j.immuni.2017.10.009 29126798

[29] Accolla RS Ramia E Tedeschi A Forlani G. CIITA-driven MHC class II expressing tumor cells as antigen presenting cell performers: toward the construction of an optimal anti-tumor vaccine. Front Immunol. (2019) 10:1806. doi: 10.3389/fimmu.2019.01806 31417570 PMC6682709

[30] Alspach E Lussier DM Miceli AP Kizhvatov I DuPage M Luoma AM. MHC-II neoantigens shape tumour immunity and response to immunotherapy. Nature. (2019) 574:696–701. doi: 10.1038/s41586-019-1671-8 31645760 PMC6858572

[31] Ferris ST Durai V Wu R Theisen DJ Ward JP Bern MD. cDC1 prime and are licensed by CD4+ T cells to induce anti-tumour immunity. Nature. (2020) 584:624–9. doi: 10.1038/s41586-020-2611-3 PMC7469755 32788723

[32] Aarntzen EH De Vries IJ Lesterhuis WJ Schuurhuis D Jacobs JF Bol K. Targeting CD4(+) T-helper cells improves the induction of antitumor responses in dendritic cell-based vaccination. Cancer Res. (2013) 73:19–29. doi: 10.1158/0008-5472.CAN-12-1127 23087058

[33] Swiecki M Colonna M. The multifaceted biology of plasmacytoid dendritic cells. Nat Rev Immunol. (2015) 15:471–85. doi: 10.1038/nri3865 PMC4808588 26160613

[34] Sichien D Scott CL Martens L Vanderkerken M Van Gassen S Plantinga M. IRF8 transcription factor controls survival and function of terminally differentiated conventional and plasmacytoid dendritic cells, respectively. Immunity. (2016) 45:626–40. doi: 10.1016/j.immuni.2016.08.013 27637148

[35] Villani AC Satija R Reynolds G Sarkizova S Shekhar K Fletcher J. Single-cell RNA-seq reveals new types of human blood dendritic cells, monocytes, and progenitors. Science. (2017) 356:eaah4573. doi: 10.1126/science.aah4573 28428369 PMC5775029

[36] Breton G Lee J Liu K Nussenzweig MC. Defining human dendritic cell progenitors by multiparametric flow cytometry. Nat Protoc. (2015) 10:1407–22. doi: 10.1038/nprot.2015.092 PMC4607256 26292072

[37] Murphy TL Murphy KM. Dendritic cells in cancer immunology. Cell Mol Immunol. (2022) 19:3–13. doi: 10.1038/s41423-021-00741-5 34480145 PMC8752832

[38] Collin M Bigley V. Human dendritic cell subsets: an update. Immunology. (2018) 154:3–20. doi: 10.1111/imm.12888 29313948 PMC5904714

[39] Reynolds G Haniffa M. Human and mouse mononuclear phagocyte networks: a tale of two species? Front Immunol. (2015) 6:330. doi: 10.3389/fimmu.2015.00330 26124761 PMC4479794

[40] Haniffa M Shin A Bigley V McGovern N Teo P See P. Human tissues contain CD141hi cross-presenting dendritic cells with functional homology to mouse CD103+ nonlymphoid dendritic cells. Immunity. (2012) 37:60–73. doi: 10.1016/j.immuni.2012.04.012 22795876 PMC3476529

[41] Granot T Senda T Carpenter DJ Matsuoka N Weiner J Gordon CL. Dendritic cells display subset and tissue-specific maturation dynamics over human life. Immunity. (2017) 46:504–15. doi: 10.1016/j.immuni.2017.02.019 PMC5415308 28329707

[42] Hildner K Edelson BT Purtha WE Diamond M Matsushita H Kohyama M. Batf3 deficiency reveals a critical role for CD8alpha+ dendritic cells in cytotoxic T cell immunity. Science. (2008) 322:1097–100. doi: 10.1126/science.1164206 PMC2756611 19008445

[43] Briseño CG Haldar M Kretzer NM Wu X Theisen DJ Wumesh K. Distinct transcriptional programs control cross-priming in classical and monocyte-derived dendritic cells. Cell Rep. (2016) 15:2462–74. doi: 10.1016/j.celrep.2016.05.025 PMC4941620 27264183

[44] Böttcher JP Bonavita E Chakravarty P Blees H Cabeza-Cabrerizo M Sammicheli S. NK cells stimulate recruitment of cDC1 into the tumor microenvironment promoting cancer immune control. Cell. (2018) 172:1022–37.e14. doi: 10.1016/j.cell.2018.01.004 29429633 PMC5847168

[45] Brass AL Kehrli E Eisenbeis CF Storb U Singh H. Pip, a lymphoid-restricted IRF, contains a regulatory domain that is important for autoinhibition and ternary complex formation with the Ets factor PU.1. Genes Dev. (1996) 10:2335–47. doi: 10.1101/gad.10.18.2335 8824592

[46] Brass AL Zhu AQ Singh H. Assembly requirements of PU.1-Pip (IRF-4) activator complexes: inhibiting function in *vivo* using fused dimers. EMBO J. (1999) 18:977–91. doi: 10.1093/emboj/18.4.977 PMC1171190 10022840

[47] Eisenbeis CF Singh H Storb U. PU.1 is a component of a multiprotein complex which binds an essential site in the murine immunoglobulin lambda 2–4 enhancer. Mol Cell Biol. (1993) 13:6452–61. doi: 10.1128/MCB.13.10.6452 PMC364704 8413244

[48] Dudziak D Kamphorst AO Heidkamp GF Buchholz VR Trumpfheller C Yamazaki S. Differential antigen processing by dendritic cell subsets in vivo. Science. (2007) 315:107–11. doi: 10.1126/science.1136080 17204652

[49] Lehmann CH Baranska A Heidkamp GF Heger L Neubert K Lühr JJ. DC subset–specific induction of T cell responses upon antigen uptake via Fcγ receptors in vivo. J Exp Med. (2017) 214:1509–28. doi: 10.1084/jem.20160951 PMC5413326 28389502

[50] Cytlak U Resteu A Pagan S Green K Milne P Maisuria S. Differential IRF8 transcription factor requirement defines two pathways of dendritic cell development in humans. Immunity. (2020) 53:353–70.e8. doi: 10.1016/j.immuni.2020.07.003 32735845 PMC7447982

[51] Bourdely P Anselmi G Vaivode K Ramos RN Missolo-Koussou Y Hidalgo S. Transcriptional and functional analysis of CD1c(+) human dendritic cells identifies a CD163(+) subset priming CD8(+)CD103(+) T cells. Immunity. (2020) 53:335–52.e8. doi: 10.1016/j.immuni.2020.06.002 32610077 PMC7445430

[52] Zhou B Lawrence T Liang Y. The role of plasmacytoid dendritic cells in cancers. Front Immunol. (2021) 12:749190. doi: 10.3389/fimmu.2021.749190 34737750 PMC8560733

[53] Di Domizio J Demaria O Gilliet M. Plasmacytoid dendritic cells in melanoma: can we revert bad into good? J Invest Dermatol. (2014) 134:1797–800. doi: 10.1038/jid.2014.155 24924760

[54] Le Mercier I Poujol D Sanlaville A Sisirak V Gobert M Durand I. Tumor promotion by intratumoral plasmacytoid dendritic cells is reversed by TLR7 ligand treatment. Cancer Res. (2013) 73:4629–40. doi: 10.1158/0008-5472.CAN-12-3058 23722543

[55] See P Dutertre C-A Chen J Günther P McGovern N Irac SE. Mapping the human DC lineage through the integration of high-dimensional techniques. Science. (2017) 356:eaag3009. doi: 10.1126/science.aag3009 28473638 PMC7611082

[56] Scandella E Men Y Legler DF Gillessen S Prikler L Ludewig B. CCL19/CCL21-triggered signal transduction and migration of dendritic cells requires prostaglandin E2. Blood. (2004) 103:1595–601. doi: 10.1182/blood-2003-05-1643 14592837

[57] Castiello L Sabatino M Jin P Clayberger C Marincola FM Krensky AM. Monocyte-derived DC maturation strategies and related pathways: a transcriptional view. Cancer Immunol Immunother. (2011) 60:457–66. doi: 10.1007/s00262-010-0954-6 PMC3086891 21258790

[58] Osugi Y Vuckovic S Hart DN. Myeloid blood CD11c+ dendritic cells and monocyte-derived dendritic cells differ in their ability to stimulate T lymphocytes. Blood J Am Soc Hematology. (2002) 100:2858–66. doi: 10.1182/blood.V100.8.2858 12351396

[59] Barry KC Hsu J Broz ML Cueto FJ Binnewies M Combes AJ. A natural killer-dendritic cell axis defines checkpoint therapy-responsive tumor microenvironments. Nat Med. (2018) 24:1178–91. doi: 10.1038/s41591-018-0085-8 PMC6475503 29942093

[60] Spranger S Bao R Gajewski TF. Melanoma-intrinsic beta-catenin signalling prevents anti-tumour immunity. Nature. (2015) 523:231–5. doi: 10.1038/nature14404 25970248

[61] Nsengimana J Laye J Filia A O’Shea S Muralidhar S Pozniak J. beta-Catenin-mediated immune evasion pathway frequently operates in primary cutaneous melanomas. J Clin Invest. (2018) 128:2048–63. doi: 10.1172/JCI95351 PMC5919828 29664013

[62] Fu C Jiang A. Dendritic cells and CD8 T cell immunity in tumor microenvironment. Front Immunol. (2018) 9:3059. doi: 10.3389/fimmu.2018.03059 30619378 PMC6306491

[63] Chow MT Ozga AJ Servis RL Frederick DT Lo JA Fisher DE. Intratumoral activity of the CXCR3 chemokine system is required for the efficacy of anti-PD-1 therapy. Immunity. (2019) 50:1498–512.e5. doi: 10.1016/j.immuni.2019.04.010 31097342 PMC6527362

[64] Spranger S Dai D Horton B Gajewski TF. Tumor-residing batf3 dendritic cells are required for effector T cell trafficking and adoptive T cell therapy. Cancer Cell. (2017) 31:711–23.e4. doi: 10.1016/j.ccell.2017.04.003 28486109 PMC5650691

[65] Garris CS Arlauckas SP Kohler RH Trefny MP Garren S Piot C. Successful anti-PD-1 cancer immunotherapy requires T cell-dendritic cell crosstalk involving the cytokines IFN-gamma and IL-12. Immunity. (2018) 49:1148–61.e7. doi: 10.1016/j.immuni.2018.09.024 30552023 PMC6301092

[66] Ruffell B Chang-Strachan D Chan V Rosenbusch A Ho CM Pryer N. Macrophage IL-10 blocks CD8+ T cell-dependent responses to chemotherapy by suppressing IL-12 expression in intratumoral dendritic cells. Cancer Cell. (2014) 26:623–37. doi: 10.1016/j.ccell.2014.09.006 PMC4254570 25446896

[67] Gerhard GM Bill R Messemaker M Klein AM Pittet MJ. Tumor-infiltrating dendritic cell states are conserved across solid human cancers. J Exp Med. (2021) 218:e20200264. doi: 10.1084/jem.20200264 33601412 PMC7754678

[68] Spranger S Luke JJ Bao R Zha Y Hernandez KM Li Y. Density of immunogenic antigens does not explain the presence or absence of the T-cell-inflamed tumor microenvironment in melanoma. Proc Natl Acad Sci U S A. (2016) 113:E7759–E68. doi: 10.1073/pnas.1609376113 PMC5137753 27837020

[69] Hegde S Krisnawan VE Herzog BH Zuo C Breden MA Knolhoff BL. Dendritic cell paucity leads to dysfunctional immune surveillance in pancreatic cancer. Cancer Cell. (2020) 37:289–307.e9. doi: 10.1016/j.ccell.2020.02.008 32183949 PMC7181337

[70] Lin JH Huffman AP Wattenberg MM Walter DM Carpenter EL Feldser DM. Type 1 conventional dendritic cells are systemically dysregulated early in pancreatic carcinogenesis. J Exp Med. (2020) 217. doi: 10.1084/jem.20190673 PMC7398166 32453421

[71] Cui Zhou D Jayasinghe RG Chen S Herndon JM Iglesia MD Navale P. Spatially restricted drivers and transitional cell populations cooperate with the microenvironment in untreated and chemo-resistant pancreatic cancer. Nat Genet. (2022) 54:1390–405. doi: 10.1038/s41588-022-01157-1 PMC9470535 35995947

[72] Cohen M Giladi A Barboy O Hamon P Li B Zada M. The interaction of CD4(+) helper T cells with dendritic cells shapes the tumor microenvironment and immune checkpoint blockade response. Nat Cancer. (2022) 3:303–17. doi: 10.1038/s43018-022-00338-5 35241835

[73] Maier B Leader AM Chen ST Tung N Chang C LeBerichel J. A conserved dendritic-cell regulatory program limits antitumour immunity. Nature. (2020) 580:257–62. doi: 10.1038/s41586-020-2134-y PMC7787191 32269339

[74] Binnewies M Mujal AM Pollack JL Combes AJ Hardison EA Barry KC. Unleashing type-2 dendritic cells to drive protective antitumor CD4(+) T cell immunity. Cell. (2019) 177:556–71.e16. doi: 10.1016/j.cell.2019.02.005 30955881 PMC6954108

[75] Duong E Fessenden TB Lutz E Dinter T Yim L Blatt S. Type I interferon activates MHC class I-dressed CD11b(+) conventional dendritic cells to promote protective anti-tumor CD8(+) T cell immunity. Immunity. (2022) 55:308–23.e9. doi: 10.1016/j.immuni.2021.10.020 34800368 PMC10827482

[76] Di Blasio S van Wigcheren GF Becker A van Duffelen A Gorris M Verrijp K. The tumour microenvironment shapes dendritic cell plasticity in a human organotypic melanoma culture. Nat Commun. (2020) 11:2749. doi: 10.1038/s41467-020-16583-0 32488012 PMC7265463

[77] James CA Baer JM Zou C Panni UY Knolhoff BL Hogg GD. Systemic alterations in type-2 conventional dendritic cells lead to impaired tumor immunity in pancreatic cancer. Cancer Immunol Res. (2023) 11:1055–67. doi: 10.1158/2326-6066.CIR-21-0946 PMC10524961 37229629

[78] Saito Y Komori S Kotani T Murata Y Matozaki T. The role of type-2 conventional dendritic cells in the regulation of tumor immunity. Cancers (Basel). (2022) 14:1976. doi: 10.3390/cancers14081976 35454882 PMC9028336

[79] Poropatich K Dominguez D Chan WC Andrade J Zha Y Wray B. OX40+ plasmacytoid dendritic cells in the tumor microenvironment promote antitumor immunity. J Clin Invest. (2020) 130:3528–42. doi: 10.1172/JCI131992 PMC7324178 32182225

[80] Sawant A Hensel JA Chanda D Harris BA Siegal GP Maheshwari A. Depletion of plasmacytoid dendritic cells inhibits tumor growth and prevents bone metastasis of breast cancer cells. J Immunol. (2012) 189:4258–65. doi: 10.4049/jimmunol.1101855 PMC3531993 23018462

[81] Dey M Chang AL Miska J Wainwright DA Ahmed AU Balyasnikova IV. Dendritic cell-based vaccines that utilize myeloid rather than plasmacytoid cells offer a superior survival advantage in Malignant glioma. J Immunol. (2015) 195:367–76. doi: 10.4049/jimmunol.1401607 PMC4475664 26026061

[82] Labidi-Galy SI Sisirak V Meeus P Gobert M Treilleux I Bajard A. Quantitative and functional alterations of plasmacytoid dendritic cells contribute to immune tolerance in ovarian cancer. Cancer Res. (2011) 71:5423–34. doi: 10.1158/0008-5472.CAN-11-0367 21697280

[83] Sisirak V Faget J Gobert M Goutagny N Vey N Treilleux I. Impaired IFN-alpha production by plasmacytoid dendritic cells favors regulatory T-cell expansion that may contribute to breast cancer progression. Cancer Res. (2012) 72:5188–97. doi: 10.1158/0008-5472.CAN-11-3468 22836755

[84] Conrad C Gregorio J Wang YH Ito T Meller S Hanabuchi S. Plasmacytoid dendritic cells promote immunosuppression in ovarian cancer via ICOS costimulation of Foxp3(+) T-regulatory cells. Cancer Res. (2012) 72:5240–9. doi: 10.1158/0008-5472.CAN-12-2271 PMC3652570 22850422

[85] Faget J Bendriss-Vermare N Gobert M Durand I Olive D Biota C. ICOS-ligand expression on plasmacytoid dendritic cells supports breast cancer progression by promoting the accumulation of immunosuppressive CD4+ T cells. Cancer Res. (2012) 72:6130–41. doi: 10.1158/0008-5472.CAN-12-2409 23026134

[86] Sharma MD Baban B Chandler P Hou DY Singh N Yagita H. Plasmacytoid dendritic cells from mouse tumor-draining lymph nodes directly activate mature Tregs via indoleamine 2,3-dioxygenase. J Clin Invest. (2007) 117:2570–82. doi: 10.1172/JCI31911 PMC1940240 17710230

[87] Challier J Bruniquel D Sewell AK Laugel B. Adenosine and cAMP signalling skew human dendritic cell differentiation towards a tolerogenic phenotype with defective CD8+ T-cell priming capacity. Immunology. (2013) 138:402–10. doi: 10.1111/imm.12053 PMC3719950 23278551

[88] Krawczyk CM Holowka T Sun J Blagih J Amiel E DeBerardinis RJ. Toll-like receptor–induced changes in glycolytic metabolism regulate dendritic cell activation. Blood. (2010) 115:4742–9. doi: 10.1182/blood-2009-10-249540 PMC2890190 20351312

[89] Novitskiy SV Ryzhov S Zaynagetdinov R Goldstein AE Huang Y Tikhomirov OY. Adenosine receptors in regulation of dendritic cell differentiation and function. Blood. (2008) 112:1822–31. doi: 10.1182/blood-2008-02-136325 PMC2518889 18559975

[90] Adamik J Munson PV Hartmann FJ Combes AJ Pierre P Krummel MF. Distinct metabolic states guide maturation of inflammatory and tolerogenic dendritic cells. Nat Commun. (2022) 13:5184. doi: 10.1038/s41467-022-32849-1 36056019 PMC9440236

[91] Ramakrishnan R Tyurin VA Veglia F Condamine T Amoscato A Mohammadyani D. Oxidized lipids block antigen cross-presentation by dendritic cells in cancer. J Immunol. (2014) 192:2920–31. doi: 10.4049/jimmunol.1302801 PMC3998104 24554775

[92] Veglia F Tyurin VA Mohammadyani D Blasi M Duperret EK Donthireddy L. Lipid bodies containing oxidatively truncated lipids block antigen cross-presentation by dendritic cells in cancer. Nat Commun. (2017) 8:2122. doi: 10.1038/s41467-017-02186-9 29242535 PMC5730553

[93] Herber DL Cao W Nefedova Y Novitskiy SV Nagaraj S Tyurin VA. Lipid accumulation and dendritic cell dysfunction in cancer. Nat Med. (2010) 16:880–6. doi: 10.1038/nm.2172 PMC2917488 20622859

[94] Giovanelli P Sandoval TA Cubillos-Ruiz JR. Dendritic cell metabolism and function in tumors. Trends Immunol. (2019) 40:699–718. doi: 10.1016/j.it.2019.06.004 31301952

[95] Møller SH Wang L Ho P-C. Metabolic programming in dendritic cells tailors immune responses and homeostasis. Cell Mol Immunol. (2022) 19:370–83. doi: 10.1038/s41423-021-00753-1 PMC8891341 34413487

[96] Pearce EJ Everts B. Dendritic cell metabolism. Nat Rev Immunol. (2015) 15:18–29. doi: 10.1038/nri3771 25534620 PMC4495583

[97] Zitvogel L Apetoh L Ghiringhelli F Kroemer G. Immunological aspects of cancer chemotherapy. Nat Rev Immunol. (2008) 8:59–73. doi: 10.1038/nri2216 18097448

[98] Preet Kaur A Alice A Crittenden MR Gough MJ. The role of dendritic cells in radiation-induced immune responses. Int Rev Cell Mol Biol. (2023) 378:61–104. doi: 10.1016/bs.ircmb.2023.02.002 37438021

[99] Panaretakis T Kepp O Brockmeier U Tesniere A Bjorklund AC Chapman DC. Mechanisms of pre-apoptotic calreticulin exposure in immunogenic cell death. EMBO J. (2009) 28:578–90. doi: 10.1038/emboj.2009.1 PMC2657583 19165151

[100] Fucikova J Becht E Iribarren K Goc J Remark R Damotte D. Calreticulin expression in human non-small cell lung cancers correlates with increased accumulation of antitumor immune cells and favorable prognosis. Cancer Res. (2016) 76:1746–56. doi: 10.1158/0008-5472.CAN-15-1142 26842877

[101] Singh-Jasuja H Hilf N Scherer HU Arnold-Schild D Rammensee HG Toes RE. The heat shock protein gp96: a receptor-targeted cross-priming carrier and activator of dendritic cells. Cell Stress Chaperones. (2000) 5:462–70. doi: 10.1379/1466-1268(2000)005<0462:THSPGA>2.0.CO;2 PMC312878 11189453

[102] Flechtner JB Cohane KP Mehta S Slusarewicz P Leonard AK Barber BH. High-affinity interactions between peptides and heat shock protein 70 augment CD8+ T lymphocyte immune responses. J Immunol. (2006) 177:1017–27. doi: 10.4049/jimmunol.177.2.1017 16818758

[103] Lehner T Wang Y Whittall T McGowan E Kelly CG Singh M. Functional domains of HSP70 stimulate generation of cytokines and chemokines, maturation of dendritic cells and adjuvanticity. Biochem Soc Trans. (2004) 32:629–32. doi: 10.1042/BST0320629 15270693

[104] Spisek R Dhodapkar MV. Towards a better way to die with chemotherapy: role of heat shock protein exposure on dying tumor cells. Cell Cycle. (2007) 6:1962–5. doi: 10.4161/cc.6.16.4601 17721082

[105] Apetoh L Ghiringhelli F Tesniere A Obeid M Ortiz C Criollo A. Toll-like receptor 4-dependent contribution of the immune system to anticancer chemotherapy and radiotherapy. Nat Med. (2007) 13:1050–9. doi: 10.1038/nm1622 17704786

[106] Dunn GP Bruce AT Ikeda H Old LJ Schreiber RD. Cancer immunoediting: from immunosurveillance to tumor escape. Nat Immunol. (2002) 3:991–8. doi: 10.1038/ni1102-991 12407406

[107] Darvin P Toor SM Sasidharan Nair V Elkord E. Immune checkpoint inhibitors: recent progress and potential biomarkers. Exp Mol Med. (2018) 50:1–11. doi: 10.1038/s12276-018-0191-1 PMC6292890 30546008

[108] Oh SA Wu DC Cheung J Navarro A Xiong H Cubas R. PD-L1 expression by dendritic cells is a key regulator of T-cell immunity in cancer. Nat Cancer. (2020) 1:681–91. doi: 10.1038/s43018-020-0075-x 35122038

[109] Lee AH Sun L Mochizuki AY Reynoso JG Orpilla J Chow F. Neoadjuvant PD-1 blockade induces T cell and cDC1 activation but fails to overcome the immunosuppressive tumor associated macrophages in recurrent glioblastoma. Nat Commun. (2021) 12:6938. doi: 10.1038/s41467-021-26940-2 34836966 PMC8626557

[110] Halpert MM Konduri V Liang D Chen Y Wing JB Paust S. Dendritic cell-secreted cytotoxic T-lymphocyte-associated protein-4 regulates the T-cell response by downmodulating bystander surface B7. Stem Cells Dev. (2016) 25:774–87. doi: 10.1089/scd.2016.0009 PMC4870609 26979751

[111] Laurent S Carrega P Saverino D Piccioli P Camoriano M Morabito A. CTLA-4 is expressed by human monocyte-derived dendritic cells and regulates their functions. Hum Immunol. (2010) 71:934–41. doi: 10.1016/j.humimm.2010.07.007 20650297

[112] Bernal-Estévez D Sánchez R Tejada RE Parra-López C. Chemotherapy and radiation therapy elicits tumor specific T cell responses in a breast cancer patient. BMC Cancer. (2016) 16:591. doi: 10.1186/s12885-016-2625-2 27484900 PMC4971722

[113] Ding Z Li Q Zhang R Xie L Shu Y Gao S. Personalized neoantigen pulsed dendritic cell vaccine for advanced lung cancer. Signal Transduct Target Ther. (2021) 6:26. doi: 10.1038/s41392-020-00448-5 33473101 PMC7817684

[114] Peng S Chen S Hu W Mei J Zeng X Su T. Combination neoantigen-based dendritic cell vaccination and adoptive T-cell transfer induces antitumor responses against recurrence of hepatocellular carcinoma. Cancer Immunol Res. (2022) 10:728–44. doi: 10.1158/2326-6066.CIR-21-0931 35476700

[115] Ingels J De Cock L Stevens D Mayer RL Théry F Sanchez GS. Neoantigen-targeted dendritic cell vaccination in lung cancer patients induces long-lived T-cells exhibiting the full differentiation spectrum. Cell Rep Med. (2024) 5:101516. doi: 10.1016/j.xcrm.2024.101516 38626769 PMC11148567

[116] Wu Q Li Q Yang L Xu H. A pilot trial of personalized neoantigen pulsed autologous dendritic cell vaccine as adjuvant treatment in hepatocellular carcinoma. J Clin Oncol. (2023) 41:e14658-e. doi: 10.1200/JCO.2023.41.16_suppl.e14658

[117] Cheever MA Higano CS. PROVENGE (Sipuleucel-T) in prostate cancer: the first FDA-approved therapeutic cancer vaccineTherapeutic prostate cancer vaccine. Clin Cancer Res. (2011) 17:3520–6. doi: 10.1158/1078-0432.CCR-10-3126 21471425

[118] Sabado RL Balan S Bhardwaj N. Dendritic cell-based immunotherapy. Cell Res. (2017) 27:74–95. doi: 10.1038/cr.2016.157 28025976 PMC5223236

[119] Kantoff PW Higano CS Shore ND Berger ER Small EJ Penson DF. Sipuleucel-T immunotherapy for castration-resistant prostate cancer. New Engl J Med. (2010) 363:411–22. doi: 10.1056/NEJMoa1001294 20818862

[120] Boettcher AN Usman A Morgans A VanderWeele DJ Sosman J Wu JD. Past, current, and future of immunotherapies for prostate cancer. Front Oncol. (2019) 9:884. doi: 10.3389/fonc.2019.00884 31572678 PMC6749031

[121] Thomsen LCV Honoré A Reisæter LAR Almås B Børretzen A Helle SI. A phase I prospective, non-randomized trial of autologous dendritic cell-based cryoimmunotherapy in patients with metastatic castration-resistant prostate cancer. Cancer Immunology Immunother. (2023) 72:2357–73. doi: 10.1007/s00262-023-03421-7 PMC10264291 36939854

[122] Vogelzang NJ Beer TM Gerritsen W Oudard S Wiechno P Kukielka-Budny B. Efficacy and safety of autologous dendritic cell–based immunotherapy, docetaxel, and prednisone vs placebo in patients with metastatic castration-resistant prostate cancer: the VIABLE phase 3 randomized clinical trial. JAMA Oncol. (2022) 8:546–52. doi: 10.1001/jamaoncol.2021.7298 PMC8832307 35142815

[123] Gilboa E Vieweg J. Cancer immunotherapy with mRNA-transfected dendritic cells. Immunol Rev. (2004) 199:251–63. doi: 10.1111/j.0105-2896.2004.00139.x 15233739

[124] Gross S Erdmann M Haendle I Voland S Berger T Schultz E. Twelve-year survival and immune correlates in dendritic cell–vaccinated melanoma patients. JCI Insight. (2017) 2:e91438. doi: 10.1172/jci.insight.91438 28422751 PMC5396520

[125] Avigan DE Vasir B George DJ Oh WK Atkins MB McDermott DF. Phase I/II study of vaccination with electrofused allogeneic dendritic cells/autologous tumor-derived cells in patients with stage IV renal cell carcinoma. J Immunother. (2007) 30:749–61. doi: 10.1097/CJI.0b013e3180de4ce8 17893567

[126] Simons JW Sacks N. Granulocyte-macrophage colony-stimulating factor-transduced allogeneic cancer cellular immunotherapy: the GVAX vaccine for prostate cancer. Urol Oncol. (2006) 24:419–24. doi: 10.1016/j.urolonc.2005.08.021 16962494

[127] Anguille S Smits EL Lion E van Tendeloo VF Berneman ZN. Clinical use of dendritic cells for cancer therapy. Lancet Oncol. (2014) 15:e257–e67. doi: 10.1016/S1470-2045(13)70585-0 24872109

[128] Wilgenhof S Corthals J Heirman C van Baren N Lucas S Kvistborg P. Phase II study of autologous monocyte-derived mRNA electroporated dendritic cells (TriMixDC-MEL) plus ipilimumab in patients with pretreated advanced melanoma. J Clin Oncol. (2016) 34:1330–8. doi: 10.1200/JCO.2015.63.4121 26926680

[129] Bol KF Aarntzen EH Hout F Schreibelt G Creemers JH Lesterhuis WJ. Favorable overall survival in stage III melanoma patients after adjuvant dendritic cell vaccination. Oncoimmunology. (2016) 5:e1057673. doi: 10.1080/2162402X.2015.1057673 26942068 PMC4760342

[130] Rodriguez J Castañón E Perez-Gracia JL Rodriguez I Viudez A Alfaro C. A randomized phase II clinical trial of dendritic cell vaccination following complete resection of colon cancer liver metastasis. J immunotherapy cancer. (2018) 6:1–7. doi: 10.1186/s40425-018-0405-z PMC6164167 30268156

[131] Liu J Zhang X Cheng Y Cao X. Dendritic cell migration in inflammation and immunity. Cell Mol Immunol. (2021) 18:2461–71. doi: 10.1038/s41423-021-00726-4 PMC8298985 34302064

[132] Mailliard RB Wankowicz-Kalinska A Cai Q Wesa A Hilkens CM Kapsenberg ML. alpha-type-1 polarized dendritic cells: a novel immunization tool with optimized CTL-inducing activity. Cancer Res. (2004) 64:5934–7. doi: 10.1158/0008-5472.CAN-04-1261 15342370

[133] Trepiakas R Pedersen AE Met O Hansen MH Berntsen A Svane IM. Comparison of alpha-Type-1 polarizing and standard dendritic cell cytokine cocktail for maturation of therapeutic monocyte-derived dendritic cell preparations from cancer patients. Vaccine. (2008) 26:2824–32. doi: 10.1016/j.vaccine.2008.03.054 18450338

[134] Okada H Kalinski P Ueda R Hoji A Kohanbash G Donegan TE. Induction of CD8+ T-cell responses against novel glioma-associated antigen peptides and clinical activity by vaccinations with {alpha}-type 1 polarized dendritic cells and polyinosinic-polycytidylic acid stabilized by lysine and carboxymethylcellulose in patients with recurrent Malignant glioma. J Clin Oncol. (2011) 29:330–6. doi: 10.1200/JCO.2010.30.7744 PMC3056467 21149657

[135] Ramanathan R Choudry H Jones H Girgis M Gooding W Kalinski P. Phase II trial of adjuvant dendritic cell vaccine in combination with celecoxib, interferon-α, and rintatolimod in patients undergoing cytoreductive surgery and hyperthermic intraperitoneal chemotherapy for peritoneal metastases. Ann Surg Oncol. (2021) 28:4637–46. doi: 10.1245/s10434-020-09464-9 PMC7784622 33400000

[136] Bol KF Schreibelt G Rabold K Wculek SK Schwarze JK Dzionek A. The clinical application of cancer immunotherapy based on naturally circulating dendritic cells. J Immunother Cancer. (2019) 7:109. doi: 10.1186/s40425-019-0580-6 30999964 PMC6471787

[137] Schreibelt G Bol KF Westdorp H Wimmers F Aarntzen EHJG Duiveman-de Boer T. Effective clinical responses in metastatic melanoma patients after vaccination with primary myeloid dendritic cells. Clin Cancer Res. (2016) 22:2155–66. doi: 10.1158/1078-0432.CCR-15-2205 26712687

[138] Tel J Aarntzen EHJG Baba T Schreibelt G Schulte BM Benitez-Ribas D. Natural human plasmacytoid dendritic cells induce antigen-specific T-cell responses in melanoma patients. Cancer Res. (2013) 73:1063–75. doi: 10.1158/0008-5472.CAN-12-2583 23345163

[139] Westdorp H Creemers JHA van Oort IM Schreibelt G Gorris MAJ Mehra N. Blood-derived dendritic cell vaccinations induce immune responses that correlate with clinical outcome in patients with chemo-naive castration-resistant prostate cancer. J Immunother Cancer. (2019) 7:302. doi: 10.1186/s40425-019-0787-6 31727154 PMC6854814

[140] de Groot WB de Wilt J Textor J Gerritsen WR De Vries J. 1078MO MIND-DC: A randomized phase III trial to assess the efficacy of adjuvant dendritic cell vaccination in comparison to placebo in stage IIIB and IIIC melanoma patients. Ann Oncol. (2020) 31:S732. doi: 10.1016/j.annonc.2020.08.1202

[141] Becker AMD Decker AH Flórez-Grau G Bakdash G Röring RJ Stelloo S. Inhibition of CSF-1R and IL-6R prevents conversion of cDC2s into immune incompetent tumor-induced DC3s boosting DC-driven therapy potential. Cell Rep Med. 5:101386. doi: 10.1016/j.xcrm.2023.101386 PMC10897516 38242119

[142] Schwarze JK Awada G Cras L Tijtgat J Forsyth R Dufait I. Intratumoral combinatorial administration of CD1c (BDCA-1)(+) myeloid dendritic cells plus ipilimumab and avelumab in combination with intravenous low-dose nivolumab in patients with advanced solid tumors: A phase IB clinical trial. Vaccines (Basel). (2020) 8:670. doi: 10.3390/vaccines8040670 33182610 PMC7712037

[143] Schwarze JK Tijtgat J Awada G Cras L Vasaturo A Bagnall C. Intratumoral administration of CD1c (BDCA-1)(+) and CD141 (BDCA-3)(+) myeloid dendritic cells in combination with talimogene laherparepvec in immune checkpoint blockade refractory advanced melanoma patients: a phase I clinical trial. J Immunother Cancer. (2022) 10:e005141. doi: 10.1136/jitc-2022-005141 36113895 PMC9486335

[144] Poulin LF Salio M Griessinger E Anjos-Afonso F Craciun L Chen J-L. Characterization of human DNGR-1+ BDCA3+ leukocytes as putative equivalents of mouse CD8α+ dendritic cells. J Exp Med. (2010) 207:1261–71. doi: 10.1084/jem.20092618 PMC2882845 20479117

[145] Wilkinson AC Ishida R Kikuchi M Sudo K Morita M Crisostomo RV. Long-term ex vivo haematopoietic-stem-cell expansion allows nonconditioned transplantation. Nature. (2019) 571:117–21. doi: 10.1038/s41586-019-1244-x PMC7006049 31142833

[146] Balan S Dalod M. In Vitro Generation of Human XCR1(+) Dendritic Cells from CD34(+) Hematopoietic Progenitors. Methods Mol Biol. (2016) 1423:19–37. doi: 10.1007/978-1-4939-3606-9_2 27142006

[147] Lee J Breton G Oliveira TYK Zhou YJ Aljoufi A Puhr S. Restricted dendritic cell and monocyte progenitors in human cord blood and bone marrow. J Exp Med. (2015) 212:385–99. doi: 10.1084/jem.20141442 PMC4354373 25687283

[148] Helft J Anjos-Afonso F van der Veen AG Chakravarty P Bonnet D e Sousa CR. Dendritic cell lineage potential in human early hematopoietic progenitors. Cell Rep. (2017) 20:529–37. doi: 10.1016/j.celrep.2017.06.075 PMC5529209 28723558

[149] Rosa FF Pires CF Kurochkin I Ferreira AG Gomes AM Palma LG. Direct reprogramming of fibroblasts into antigen-presenting dendritic cells. Sci Immunol. (2018) 3:eaau4292. doi: 10.1126/sciimmunol.aau4292 30530727

[150] Maraskovsky E Brasel K Teepe M Roux ER Lyman SD Shortman K. Dramatic increase in the numbers of functionally mature dendritic cells in Flt3 ligand-treated mice: multiple dendritic cell subpopulations identified. J Exp Med. (1996) 184:1953–62. doi: 10.1084/jem.184.5.1953 PMC2192888 8920882

[151] Salmon H Idoyaga J Rahman A Leboeuf M Remark R Jordan S. Expansion and activation of CD103+ dendritic cell progenitors at the tumor site enhances tumor responses to therapeutic PD-L1 and BRAF inhibition. Immunity. (2016) 44:924–38. doi: 10.1016/j.immuni.2016.03.012 PMC4980762 27096321

[152] Lynch DH Andreasen A Maraskovsky E Whitmore J Miller RE Schuh JC. Flt3 ligand induces tumor regression and antitumor immune responses in vivo. Nat Med. (1997) 3:625–31. doi: 10.1038/nm0697-625 9176488

[153] Chen K Braun S Lyman S Fan Y Traycoff CM Wiebke EA. Antitumor activity and immunotherapeutic properties of Flt3-ligand in a murine breast cancer model. Cancer Res. (1997) 57:3511–6. 9270021

[154] Esche C Subbotin VM Maliszewski C Lotze MT Shurin MR. FLT3 ligand administration inhibits tumor growth in murine melanoma and lymphoma. Cancer Res. (1998) 58:380–3. 9458075

[155] Morse MA Nair S Fernandez-Casal M Deng Y St Peter M Williams R. Preoperative mobilization of circulating dendritic cells by Flt3 ligand administration to patients with metastatic colon cancer. J Clin Oncol. (2000) 18:3883–93. doi: 10.1200/JCO.2000.18.23.3883 11099317

[156] Rini BI Paintal A Vogelzang NJ Gajewski TF Stadler WM. Flt-3 ligand and sequential FL/interleukin-2 in patients with metastatic renal carcinoma: clinical and biologic activity. J Immunother. (2002) 25:269–77. doi: 10.1097/00002371-200205000-00010 12000869

[157] Disis ML Rinn K Knutson KL Davis D Caron D dela Rosa C. Flt3 ligand as a vaccine adjuvant in association with HER-2/neu peptide-based vaccines in patients with HER-2/neu–overexpressing cancers. Blood J Am Soc Hematology. (2002) 99:2845–50. doi: 10.1182/blood.V99.8.2845 11929774

[158] Bhardwaj N Friedlander PA Pavlick AC Ernstoff MS Gastman BR Hanks BA. Flt3 ligand augments immune responses to anti-DEC-205-NY-ESO-1 vaccine through expansion of dendritic cell subsets. Nat Cancer. (2020) 1:1204–17. doi: 10.1038/s43018-020-00143-y 35121932

[159] Umemura Y Orringer D Junck L Varela ML West MEJ Faisal SM. Combined cytotoxic and immune-stimulatory gene therapy for primary adult high-grade glioma: a phase 1, first-in-human trial. Lancet Oncol. (2023) 24:1042–52. doi: 10.1016/S1470-2045(23)00347-9 37657463

[160] Cueto FJ Sancho D. The Flt3L/Flt3 axis in dendritic cell biology and cancer immunotherapy. Cancers. (2021) 13:1525. doi: 10.3390/cancers13071525 33810248 PMC8037622

[161] Bennett SRM Carbone FR Karamalis F Flavell RA Miller JFAP Heath WR. Help for cytotoxic-T-cell responses is mediated by CD40 signalling. Nature. (1998) 393:478–80. doi: 10.1038/30996 9624004

[162] Byrne Katelyn T Vonderheide Robert H. CD40 stimulation obviates innate sensors and drives T cell immunity in cancer. Cell Rep. (2016) 15:2719–32. doi: 10.1016/j.celrep.2016.05.058 PMC4917417 27292635

[163] French RR Chan HTC Tutt AL Glennie MJ. CD40 antibody evokes a cytotoxic T-cell response that eradicates lymphoma and bypasses T-cell help. Nat Med. (1999) 5:548–53. doi: 10.1038/8426 10229232

[164] van Mierlo GJD den Boer AT Medema JP van der Voort EIH Fransen MF Offringa R. CD40 stimulation leads to effective therapy of CD40− tumors through induction of strong systemic cytotoxic T lymphocyte immunity. Proc Natl Acad Sci. (2002) 99:5561–6. doi: 10.1073/pnas.082107699 PMC122809 11929985

[165] Oba T Hoki T Yamauchi T Keler T Marsh HC Cao X. A critical role of CD40 and CD70 signaling in conventional type 1 dendritic cells in expansion and antitumor efficacy of adoptively transferred tumor-specific T cells. J Immunol. (2020) 205:1867–77. doi: 10.4049/jimmunol.2000347 PMC7511447 32848036

[166] Eliopoulos AG Davies C Knox PG Gallagher NJ Afford SC Adams DH. CD40 induces apoptosis in carcinoma cells through activation of cytotoxic ligands of the tumor necrosis factor superfamily. Mol Cell Biol. (2000) 20:5503–15. doi: 10.1128/MCB.20.15.5503-5515.2000 PMC86001 10891490

[167] Funakoshi S Longo D Beckwith M Conley D Tsarfaty G Tsarfaty I. Inhibition of human B-cell lymphoma growth by CD40 stimulation. Blood. (1994) 83:2787–94. doi: 10.1182/blood.V83.10.2787.2787 7514045

[168] Charpentier M Formenti S Demaria S. CD40 agonism improves anti-tumor T cell priming induced by the combination of radiation therapy plus CTLA4 inhibition and enhances tumor response. Oncoimmunology. (2023) 12:2258011. doi: 10.1080/2162402X.2023.2258011 37727740 PMC10506429

[169] Oba T Long MD Keler T Marsh HC Minderman H Abrams SI. Overcoming primary and acquired resistance to anti-PD-L1 therapy by induction and activation of tumor-residing cDC1s. Nat Commun. (2020) 11:5415. doi: 10.1038/s41467-020-19192-z 33110069 PMC7592056

[170] Shankara Narayanan JS Hayashi T Erdem S McArdle S Tiriac H Ray P. Treatment of pancreatic cancer with irreversible electroporation and intratumoral CD40 antibody stimulates systemic immune responses that inhibit liver metastasis in an orthotopic model. J Immunother Cancer. (2023) 11:e006133. doi: 10.1136/jitc-2022-006133 36634919 PMC9843215

[171] Yamauchi T Hoki T Oba T Kajihara R Attwood K Cao X. CD40 and CD80/86 signaling in cDC1s mediate effective neoantigen vaccination and generation of antigen-specific CX3CR1(+) CD8(+) T cells. Cancer Immunol Immunother. (2022) 71:137–51. doi: 10.1007/s00262-021-02969-6 PMC8715856 34037810

[172] Tan TJ Ang WXG Wang WW Chong HS Tan SH Cheong R. A phase I study of an adenoviral vector delivering a MUC1/CD40-ligand fusion protein in patients with advanced adenocarcinoma. Nat Commun. (2022) 13:6453. doi: 10.1038/s41467-022-33834-4 36307410 PMC9616917

[173] De Keersmaecker B Claerhout S Carrasco J Bar I Corthals J Wilgenhof S. TriMix and tumor antigen mRNA electroporated dendritic cell vaccination plus ipilimumab: link between T-cell activation and clinical responses in advanced melanoma. J Immunother Cancer. (2020) 8:e000329. doi: 10.1136/jitc-2019-000329 32114500 PMC7057443

[174] Vonderheide RH. CD40 agonist antibodies in cancer immunotherapy. Annu Rev Med. (2020) 71:47–58. doi: 10.1146/annurev-med-062518-045435 31412220

[175] Weiss SA Sznol M Shaheen M Berciano-Guerrero M Couselo EM Rodríguez-Abreu D. A phase II trial of the CD40 agonistic antibody sotigalimab (APX005M) in combination with nivolumab in subjects with metastatic melanoma with confirmed disease progression on anti-PD-1 therapy. Clin Cancer Res. (2024) 30:74–81. doi: 10.1158/1078-0432.CCR-23-0475 37535056 PMC10767304

[176] Padrón LJ Maurer DM O’Hara MH O’Reilly EM Wolff RA Wainberg ZA. Sotigalimab and/or nivolumab with chemotherapy in first-line metastatic pancreatic cancer: clinical and immunologic analyses from the randomized phase 2 PRINCE trial. Nat Med. (2022) 28:1167–77. doi: 10.1038/s41591-022-01829-9 PMC9205784 35662283

[177] Salomon R Dahan R. Next generation CD40 agonistic antibodies for cancer immunotherapy. Front Immunol. (2022) 13. doi: 10.3389/fimmu.2022.940674 PMC9326085 35911742

[178] Matsuo K Kitahata K Kawabata F Kamei M Hara Y Takamura S. A highly active form of XCL1/lymphotactin functions as an effective adjuvant to recruit cross-presenting dendritic cells for induction of effector and memory CD8+ T cells. Front Immunol. (2018) 9:2775. doi: 10.3389/fimmu.2018.02775 30542351 PMC6277777

[179] Lahoud M Radford K. Enhancing the immunogenicity of cancer vaccines by harnessing CLEC9A. Hum Vaccines immunotherapeutics. (2022) 18:1873056. doi: 10.1080/21645515.2021.1873056 PMC8920153 33625943

[180] Gou S Wang S Liu W Chen G Zhang D Du J. Adjuvant-free peptide vaccine targeting Clec9a on dendritic cells can induce robust antitumor immune response through Syk/IL-21 axis. Theranostics. (2021) 11:7308. doi: 10.7150/thno.56406 34158852 PMC8210616

[181] Thacker EE Nakayama M Smith BF Bird RC Muminova Z Strong TV. A genetically engineered adenovirus vector targeted to CD40 mediates transduction of canine dendritic cells and promotes antigen-specific immune responses in vivo. Vaccine. (2009) 27:7116–24. doi: 10.1016/j.vaccine.2009.09.055 PMC2784276 19786146

[182] Sharma PK Dmitriev IP Kashentseva EA Raes G Li L Kim SW. Development of an adenovirus vector vaccine platform for targeting dendritic cells. Cancer Gene Ther. (2018) 25:27–38. doi: 10.1038/s41417-017-0002-1 29242639 PMC5972836

[183] Gao J-Q Sugita T Kanagawa N Iida K Okada N Mizuguchi H. Anti-tumor responses induced by chemokine CCL19 transfected into an ovarian carcinoma model via fiber-mutant adenovirus vector. Biol Pharm Bulletin. (2005) 28:1066–70. doi: 10.1248/bpb.28.1066 15930746

[184] Sanchez-Lugo YE Perez-Trujillo JJ Gutierrez-Puente Y Garcia-Garcia A Rodriguez-Rocha H Barboza-Quintana O. CXCL10/XCL1 fusokine elicits in *vitro* and in *vivo* chemotaxis. Biotechnol letters. (2015) 37:779–85. doi: 10.1007/s10529-014-1746-4 25515795

[185] Botelho NK Tschumi BO Hubbell JA Swartz MA Donda A Romero P. Combination of synthetic long peptides and XCL1 fusion proteins results in superior tumor control. Front Immunol. (2019) 10:294. doi: 10.3389/fimmu.2019.00294 30863405 PMC6399421

[186] Yarema KJ Mahal LK Bruehl RE Rodriguez EC Bertozzi CR. Metabolic delivery of ketone groups to sialic acid residues: Application to cell surface glycoform engineering. J Biol Chem. (1998) 273:31168–79. doi: 10.1074/jbc.273.47.31168 9813021

[187] Prescher JA Dube DH Bertozzi CR. Chemical remodelling of cell surfaces in living animals. Nature. (2004) 430:873–7. doi: 10.1038/nature02791 15318217

[188] Kim J Li WA Choi Y Lewin SA Verbeke CS Dranoff G. Injectable, spontaneously assembling, inorganic scaffolds modulate immune cells in *vivo* and increase vaccine efficacy. Nat Biotechnol. (2015) 33:64–72. doi: 10.1038/nbt.3071 25485616 PMC4318563

[189] Li AW Sobral MC Badrinath S Choi Y Graveline A Stafford AG. A facile approach to enhance antigen response for personalized cancer vaccination. Nat materials. (2018) 17:528–34. doi: 10.1038/s41563-018-0028-2 PMC5970019 29507416

[190] Heras-Murillo I Adán-Barrientos I Galán M Wculek SK Sancho D. Dendritic cells as orchestrators of anticancer immunity and immunotherapy. Nat Rev Clin Oncol. (2024) 21:257–77. doi: 10.1038/s41571-024-00859-1 38326563

[191] van Willigen WW Bloemendal M Gerritsen WR Schreibelt G de Vries IJM Bol KF. Dendritic cell cancer therapy: vaccinating the right patient at the right time. Front Immunol. (2018) 9. doi: 10.3389/fimmu.2018.02265 PMC6174277 30327656

